# Phylogenetic prospecting for cryptic species of the genus Merluccius (Actinopterygii: Merlucciidae)

**DOI:** 10.1038/s41598-021-85008-9

**Published:** 2021-03-15

**Authors:** Montse Pérez, María Fernández-Míguez, Jesús Matallanas, Domingo Lloris, Pablo Presa

**Affiliations:** 1 AquaCOV, Centro Oceanográfico de Vigo, Instituto Español de Oceanografía, 36390 Vigo, Spain; 2 Laboratorio de Recursos Genéticos Marinos, Facultad de Biología, CIM-Universidad de Vigo, 36310 Vigo, Spain; 3 Unidad de Zoología, Departamento de Biología Animal, Biología Vegetal y Ecología, Universidad Autónoma de Barcelona, 08193 Barcelona, Spain; 4 Institut de Ciències del Mar (CMIMA-CSIC), 08003 Barcelona, Spain

**Keywords:** Haplotypes, Speciation, Taxonomy

## Abstract

Hakes of the genus *Merluccius* include 11 valid species as well a number of rare morphotypes suspected to be “cryptic species”. Concatenated nucDNA ITS1-rDNA and mtDNA cyt b sequences plus nested ITS1Nes sequences allowed to ascribe 14 specimens of nine rare morphotypes from the South Pacific and the South Atlantic to the phylogenetic backbone of this genus. Bayesian analyses pointed to *M. bilinearis* and *M. albidus* as the oldest species of the genus and the New World cluster, respectively. The phylogenetic status of *M. angustimanus* from the upper Gulf of California suggests its hybrid origin between *M. gayi* and *M. productus* from about 0.25 MYA, although an ever since confinement of a subset of those species cannot be ruled out. The molecular phylodiagnostic test suggests a common origin of all rare morphotypes and the absence of cryptic hake species in the Southern Cone. The molecular background of the morphotypes distributed between the Western Pacific South of New Zealand and the western Atlantic South of Argentina is compatible with their hybrid origin between *M. gayi* and both, *M. australis* or *M. hubbsi*, respectively.

## Introduction

The genus *Merluccius* comprises 11 valid species that occur on most temperate and tropical continental shelves except the Asian shores of the Pacific Ocean^1^. Hakes show an anti-tropical distribution in the Atlantic Ocean and the Eastern Pacific and a latitudinal bathymetric overlap between isotherms 7 °C and 23 °C^2–4^. Based on osteological data^5,6^ it is believed that genus *Palaeogadus* as ancestor of genus *Merluccius*, arose near Greenland in the early Eocene (*ca*. 50 MYA)^7^, dispersed southwards along the North American and Eurasian shelves and entered the Pacific^8^. The earliest known merluccid fossils date back to the Middle Oligocene (*ca*. 27–33 MYA) in a large inland sea that covered much of central Europe and connected to a temperate Arctic Ocean^9^. It is believed that either an ancestral species of *Merluccius* or the extant *M. bilinearis* experienced an evolutionary radiation in two superclusters, i.e. Old World hakes (the Euro-African supercluster) and New World hakes (the American supercluster)^5–8^. Also, it is hypothesized that a widening rift between Europe and North America plates prompted vicariant speciation and that recurrent dispersal events and adaptation to temperature regimes also played a role in the speciation of this genus^10^. Subsequent geological events such as the closure of the Panama Seaway over 3.5 MYA acted as a geographical barrier between Atlantic and Pacific lineages^8,11^. Successive population fragmentation and expansion due to climatic oscillations during Pleistocene glaciations allowing founder phenomena cannot be ruled out^12^. Such origin and dispersal hypotheses are congruent with the actual phylogeny of the genus worked out after parasite data^8^ morphology^3,5,6,13^ and genetic data^14–16^.

**Figure 1:**
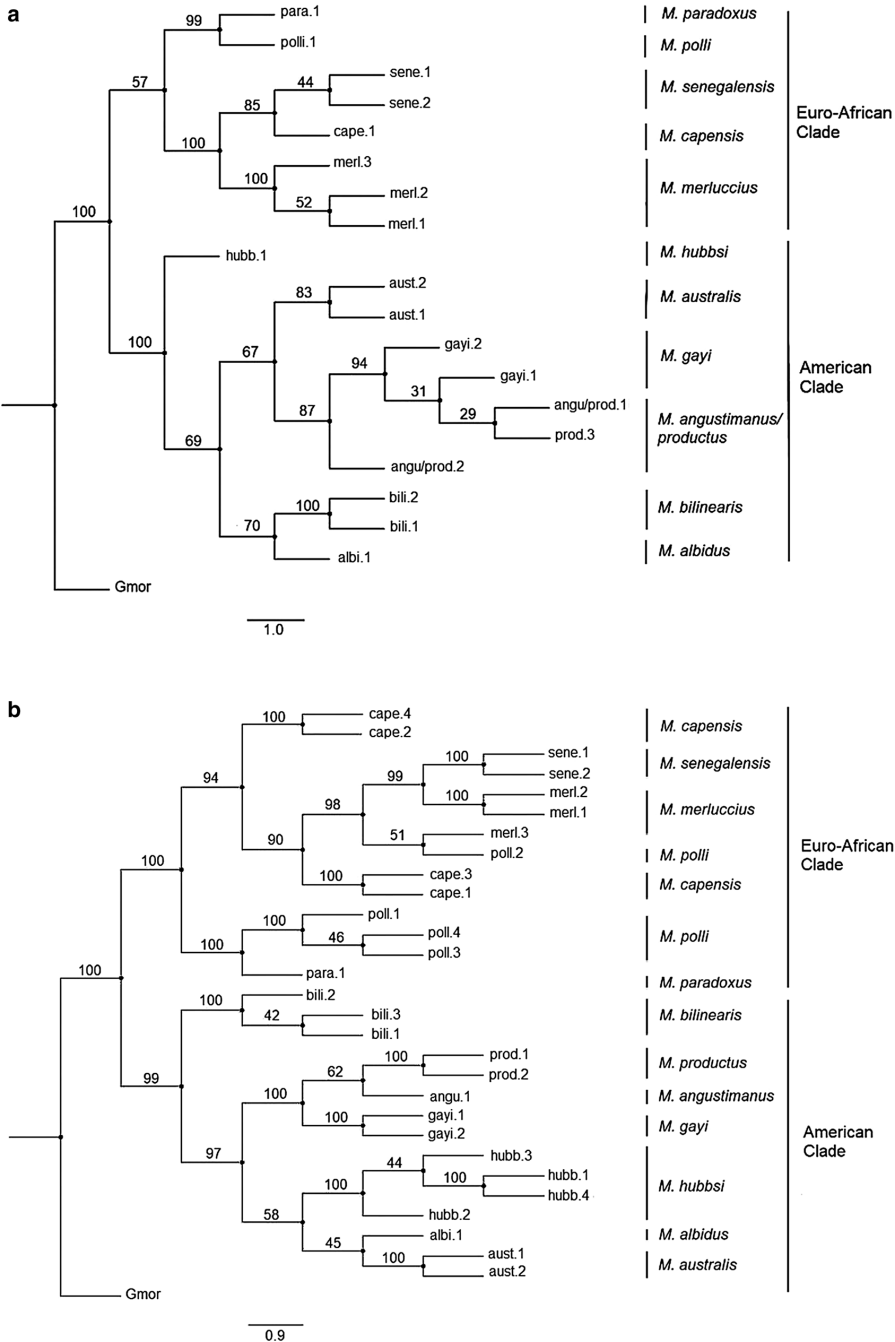
BEAST v1.8.4^85^ phylogenetic reconstruction of genus *Merluccius* spp. using Bayesian inference on both, (**a**) the substitution model HKY85 + I + G on ITS1 variants and (**b**) the substitution model GTR + G on cyt b haplotypes. Percentage of trees over 5000 bootstrap replicates are shown above branches. OTU Gmor corresponds to the outgroup *Gadus morhua*. Scale bar indicates the No. of nucleotide substitutions per 100 DNA residues. Sample codes are given in Table 1 and are followed either by the number of ITS1 variants per species (Fig. 1a and Supp. Table S3) or by the number of cyt b haplotypes per species (Fig. 1b and Supp. Table S4).

The Old World supercluster comprises five well-defined species and with occasional hybrids between sympatric species, e.g. *M. capensis* x *M. paradoxus*^17^. The New World supercluster comprises three clusters of two species each, an Atlantic north cluster that comprises *M. bilinearis* and *M. albidus,* a southern cluster that comprises *M. hubbsi* and *M. australis*, and a Pacific cluster that groups *M. gayi* and *M. productus*. Molecular systematics of *Merluccius* generally distinguishes those 11 species^18^, however there are still knowledge gaps in hake taxonomy as some specimens found in regions of species overlap show significant morphological divergence from extant species^13^. For instance, the phylogenetic relationships within the New World supercluster have been repeatedly interrogated due to the uncertain taxonomic status of some pairs of morphotypes such as *M. gayi gayi*^19^ *vs. M. gayi peruanus*^20^, *M. angustimanus*^21^ *vs*. *M. hernandezi*^22^, *M. hubbsi vs. M. patagonicus*^23^ or *M. australis* vs. *M. polylepis* or *M. tasmanicus*^24^. Several of those morphotypes have been recently taken as synonymous with extant species. For instance, the three stocks of *M. productus* believed to exist in the northeast Pacific corridor from Washington State to Costa Rica^25,26^ likely belong to a single hake species^27,28^. The high variability and overlap of meristic traits between *M. hernandezi* and *M. angustimanus* suggests they are synonymous forms^13^ and that *M. angustimanus* is a subpopulation of *M. productus* confined to the northern Gulf of California^27^. Also, meristic (see Fig. 1 from^23^) and molecular analyses^29,30^ showed that *M. patagonicus* could be synonymous with *M. hubbsi* and that *M. tasmanicus* could be synonymous with *M. australis*^31^. Taxonomic uncertainties are expected in closely-related species that show extensive overlap in morphological and meristic traits. Resolution of those questions has been complicated by the small number of morphotype samples available, the lack of a systematic sampling plan over a reasonable number of localities and the choice of the most appropriate phylogenetic reconstruction algorithm.

We address those issues with the most comprehensive sample collection yet made in this genus, which comprises all 11 valid species^13^ as well as *M. angustimanus* from the Gulf of California^21^ and 14 specimens of nine rare morphotypes classified as *M. tasmanicus* from New Zealand waters^23^, *M. polylepis* from the Pacific coast of Chile^32^, *M. patagonicus* from the Atlantic South^23^, uncatalogued specimens of *M. hubbsi* from the Beagle Channel and Puerto Madryn in Argentina^23^, and rare specimens of *M. australis* from Chile^23^ and New Zealand^24^. An integrative multimarker phylogenetic reconstruction of genus *Merluccius* is enforced to determine both, the phylogenetic congruence and synergy between mtDNA (cyt b) and nucDNA (ITS1) sequences for inference of phylogenetic relationships in this genus and the genetic prospecting for cryptic species within the New World supercluster, as suspected on the rare morphotypes described so far.

## Results

### Description of samples and sequences

We examined 1205 specimens from 11 valid hake species and *M. angustimanus* (Table 1) as well as nine morphotypes (Table 2). The aligned ITS1 region had a length of 692 bp and comprised the ITS1-rDNA sequence, 53 bp from the 3′-end of the 18S-rDNA gene and 20 bp from the 5′-end of the 5.8S-rDNA gene^33^. A total of 254 variable sites were found among 85 specimens from 12 hake species (including *M. angustimanus* as putative species) (Supp. Table S1). The ITS1 sequences showed similar %GC content between species and a general low transversion rate (Supp. Table S2). The full dataset of 85 ITS1 sequences comprised 19 variants (Supp. Table S3). The aligned cyt b region had a length of 465 bp and comprised 428 bp from the 5′-end of the cyt b and 37 bp from the 3′-end of the mitochondrial DNA gene tRNA-Glu. A total of 129 variable sites were found among 66 specimens from 12 hake species as including *M. angustimanus* (Supp. Table S1). The 66 cyt b sequences comprised 29 haplotypes as one to four per species (Supp. Table S4). *M. angustimanus* showed a single haplotype (HakeCytb.18) that was molecularly close to those of *M. productus, M. gayi* and *M. albidus*. Concatenated sequence information from both DNA regions was available on 42 specimens, comprised 1158 bp in length with 381 variable sites (Supp. Table S1) and identified 25 variants (Supp. Figure S4). The aligned ITS1Nes sequences from 39 specimens of reference and 46 clones from 14 specimens of nine morphotypes had a length of 66 bp and 13 variable sites (Supp. Table S1). Those 85 ITS1Nes sequences comprised 11 variants (HakeITS1Nes.1–11) (Table 3). HakeITS1Nes.2 was shared among the Pacific species and four morphotypes, HakeITS1Nes.5 was shared between the Atlantic North species, HakeITS1Nes.6 was shared among all morphotypes and HakeITS1Nes.9 was shared between two morphotypes (Table 3). All the morphotypes shared at least one ITS1Nes variant among each other or with known species, but also exhibited specific variants, e.g. HakeITS1Nes.7-8-10-11. The recombination parameter (*R*) detected three pairs of sites in the ITS1Nes region with at least one recombination event^34^, i.e. between sites 5–8, 8–14 and 14–56.

**Table 1:** Origin of hakes used in reconstructing the phylogenetic backbone of genus *Merluccius* spp. N is the number of specimens sampled per location.

Hake species	Code	Common names	Latitude	Location	Coordinates	N
*Merluccius merluccius*	merl	European hake	21–62ºN	Spain Italy United Kingdom	37º35′N/08º50′W 38º03′N/12º56′E 55º30′N/04º36′E	186 36 55
*Merluccius senegalensis*	sene	Senegalese hake	10–33ºN	Senegal Namibia Mauritania	15º01′N/18º00′W 18º10′N/16º20′W 21º40′N/17º55′W	7 25 50
*Merluccius polli*	poll	Benguela hake	20º N–19ºS	Senegal Mauritania Spain-Morocco	15º01′N/18º00′W 19º37′N/7º06′W 27º15′N/14º10′W	2 20 100
*Merluccius capensis*	cape	Shallow-water Cape hake	00–34ºS	Namibia South Africa	24º10′S/14º23′E 25º33′S/15º13′E	63 20
*Merluccius paradoxus*	para	Deep-water Cape hake	22–34ºS	South Africa South Africa	25º33′S/15º13′E 34º10′S/17º10′E	68 83
*Merluccius productus*	prod	Pacific hake	25–51ºN	Canada Canada Canada	48º08′N/122º20′W 49º10′N/123º10′W 50º00′N/125º06′W	30 45 10
*Merluccius gayi*	gayi	Peruvian hake Chilean hake	03–47ºS	Peru–Chile Chile Chile	08º50′S/80º00′W 24º40′S/70º50′W 30º00′S/71º55′W	8 21 89
*Merluccius australis*	aust	Antarctic queen hake New Zealand hake	40–57ºS	Chile Australia	41º20′S/74º35′W 43º40′S/169º25′E	60 23
*Merluccius hubbsi*	hubb	Argentine hake	25–54ºS	Argentina Argentina	46º30′S/60º45′W 48º30′S/61º30′W	37 70
*Merluccius albidus*	albi	Offshore hake	20–35ºN	United States United States	35º21′N/70º50′W 37º21′N/73º33′W	4 5
*Merluccius bilinearis*	bili	Silver hake	36–47ºN	United States United States United States	39º00′N/73º10′W 40º40′N/72º00′W 42º30′N/68º33′W	6 6 60
Merluccius angustimanus*	angu	Panama hake	05–23ºN	Mexico	29º50′N/113º20′W	6
**Outgroup taxon**						
*Gadus morhua*	Gmor	Atlantic cod	36–37ºN	United States	37º21′N/73º33′W	10

*The so-called *M. hernandezi* (California hake) in^33^.

**Table 2:** Origin of rare hake morphotypes from directed sampling campaigns and museum holotypes and paratypes used in the molecular phylodiagnosis. N is the number of specimens analyzed per morphotype (see also Supp. Table S9).

Morphotypes	Code	References	Location	Coordinates	N
*M. tasmanicus*	TASM	Holotype NMNZ P.5566^24^	Tasman Bay, New Zealand	40º52′S/173º08′E	1
*M. tasmanicus*	TASM	Paratype NMNZ P.3963^24^	Cook Strait, New Zealand	41º30′S174º30′E	1
*M. patagonicus*	PATA	Paratypes IIPB 501–504/2001^23^	Comodoro Ribadavia, Argentina	45º30′S/65º30′W	3
*M. polylepis*	POLY	Holotype^32^	Chiloé, Chile	41º20′S/74º35′W	1
*M. polylepis*	POLY	Paratype USNM 157765^23^	Puerto Montt, Chile	41º57′S/72º87′W	1
*M. hubbsi*	HUBB	Paratype IIPD 92/1987^23^	Beagle Channel, Argentina	54°48.9′S/68°14.8′W	1
*M. hubbsi*	HUBB	Uncatalogued juveniles^23^ (*cf.* A. E. Ruiz and R. R. Fontdacaro)	Puerto Madryn, Argentina	43°50′S/65°02′W	2
*M. australis*	AUST	Paratypes MOVI 27492–27493, formerly NMNZ P.13122^24^	Chalkey Intel, Fiordland, New Zealand	46º03′S/166º20′E	2
*M. australis*	AUST	Two uncatalogued juveniles^23^ (*cf.* R. Bravo)	Aysén, Chile	46º22′S/ 75º27′W	2

**Table 3:** Absolute frequency of ITS1Nes variants per species and morphotypes and their polymorphic sites generated with DnaSP v5.10.1^77^. Alpha-numeric codes for ITS1Nes variants are shown ordinal as distinct from full ITS1 variants. Specimen codes are given in Tables 1 and 2.

Variants	Hake species								Hake morphotypes					Polymorphic sites	
	merl	prod	gayi	angu	aust	hubb	bili	albi	AUST	TASM	POLY	PATA	HUBB	112355 358242656	5566 8926
HakeITS1Nes.1	1	–	–	–	–	–	–	–	–	–	–	–	–	TTTTTGACT	ACGT
HakeITS1Nes.2	–	10	3	3	–	–	–	–	1	2	2	–	2	.C.CC..AC	.GA
HakeITS1Nes.3	–	–	–	–	8	–	–	–	–	–	–	–	–	.CC.C..AC	.GA
HakeITS1Nes.4	–	–	–	–	–	5	–	–	–	–	–	–	5	.CC.C.GAC	.GAC
HakeITS1Nes.5	–	–	–	–	–	–	6	3	–	–	–	–	–	.CCC…A	.GA
HakeITS1Nes.6	–	–	–	–	–	–	–	–	6	5	4	1	12	.C.CC.GAC	.GA
HakeITS1Nes.7	–	–	–	–	–	–	–	–	–	–	–	–	1	CC.CC.GAC	.GA
HakeITS1Nes.8	–	–	–	–	–	–	–	–	–	–	–	1	–	.CCCC.GAC	.GA
HakeITS1Nes.9	–	–	–	–	–	–	–	–	1	–	1	–	–	.CCCC..AC	.GA
HakeITS1Nes.10	–	–	–	–	–	–	–	–	–	–	–	1	–	.C.GC.GAC	GGA
HakeITS1Nes.11	–	–	–	–	–	–	–	–	–	–	1	–-	–	.C.CCCGAC	.GA

### Nucleotide divergence and genetic distance

The lowest average number of nucleotide substitutions per site among ITS1 sequences (*D*_xy_ = 0.004–0.009) as well as among cyt b sequences (*D*_xy_ = 0.007–0.014) were observed in pairwise comparisons of *M. angustimanus*, *M. productus* and *M. gayi* (Supp. Table S5; Supp. Table S6). *M. gayi* showed a similar evolutionary divergence of ITS1 (*d* = 0.004) and cyt b (*d* = 0.008–0.010) from both, *M. angustimanus* and *M. productus*, respectively. Those latter species showed the lowest pairwise divergence in the dataset, i.e. for ITS1 (*d* = 0.001) (Supp. Table S5) as well as for cyt b (*d* = 0.005) (Supp. Table S6). The divergence pattern after ITS1Nes sequences was less than that observed on full sequences of ITS1 or cyt b because of the lower number of variable sites in the former, and *M. angustimanus*-*M. productus-M. gayi* did not diverge among each other (Supp. Table S7). The average number of nucleotide substitutions per site (*D*_xy_) (Table 4a, Supp. Table S8a) and the net evolutionary divergence between species (Table 4b, Supp. Table S8b) from ITS1Nes showed that the morphotype-specific sequences of *M. patagonicus* (PATA), *M. tasmanicus* (TASM), *M. polylepis* (POLY), *M. hubbsi* (TASM) and *M. australis* (AUST) were more similar to Pacific hakes than to any other species in the New World supercluster (see also ITS1Nes variation in Table 3).

**Table 4:** (a) Average number of nucleotide substitutions per site (*D*_xy_) between New World hakes (three clusters) and hake morphotypes under test (in capitals) as generated with DnaSP v5.10.1^77^, (b) Estimates of the Net Evolutionary Divergence (*d*) between New World hakes and hake morphotypes as generated with MEGA v7.0.20^76^. Both estimates were computed on ITS1Nes sequences and used the European hake (merl) as a relative measure of divergence.

	Pacific cluster				Austral cluster			Atlantic North cluster		
	prod	gayi	angu	$\stackrel{-}{x}$±*S.D.* (*CI*)^1^	aust	hubb	$\stackrel{-}{x}$±*S.D.* (*CI*)^2^	bili	albi	$\stackrel{-}{x}$± *S.D.*(*CI*)^3^
**a**										
AUST	0.016	0.016	0.016	0.027 ± 0.006 [0.023, 0.030]	0.008	0.047	0.044 ± 0.016 [0.035, 0.055]	0.080	0.076	0.073** ± **0.009 [0.067, 0.078]
TASM	0.031	0.031	0.032		0.055	0.065		0.067	0.060	
POLY	0.033	0.033	0.034		0.053	0.064		0.070	0.063	
PATA	0.031	0.031	0.032		0.047	0.046		0.089	0.085	
HUBB	0.028	0.028	0.028		0.054	0.061		0.075	0.068	
merl	0.111	0.111	0.115		0.113	0.143		0.117	0.115	
**b**										
AUST	0.009	0.009	0.009	0.009 ± 0.003 [0.007, 0.010]	0.034	0.037	0.034 ± 0.006 [0.031, 0.038]	0.070	0.071	0.053** ± **0.016 [0.043, 0.063]
TASM	0.007	0.007	0.008		0.041	0.042		0.040	0.037	
POLY	0.007	0.007	0.008		0.026	0.038		0.041	0.038	
PATA	0.014	0.014	0.015		0.029	0.028		0.076	0.076	
HUBB	0.008	0.008	0.009		0.032	0.040		0.053	0.050	
merl	0.128	0.128	0.134		0.131	0.182		0.135	0.144	

^1^*D*_xy_ average (**a**) or *d* (**b**) on pairwise comparisons between Pacific hakes (*M. productus*, *M. gayi* and *M. angustimanus*) and hake morphotypes (in capitals).

^2^*D*_xy_ average (**a**) or* d* (**b**) on pairwise comparisons between Austral hakes (*M. australis* and *M. hubbsi*) and hake morphotypes (in capitals).

^3^*D*_xy_ average (**a**) or* d* (**b**) on pairwise comparisons between Atlantic North hakes (*M. bilinearis* and *M. albidus*) and hake morphotypes (in capitals).

### Clustering methods

The major groups of species as inferred from the Euclidean divergence of the PCoA correctly identified a) the two major complexes of hakes, Old World *versus* New World hakes (Supp. Figure S1a, b) Atlantic *versus* Pacific New World hakes (Supp. Figure S1b, and c) the closeness of hake morphotypes to both, the Pacific group and the Austral group (Supp. Figure S1c). The AMOVA agreed with the partition among the groups identified by PCoA using ITS1 variants, i.e. Old World/ New World and Atlantic New World /Pacific New World /Austral New World (Table 5). Noteworthy, the joint analysis including the morphotypes in the Pacific cluster produced the largest within cluster variation as compared to any other hierarchical level.

**Table 5:** Analysis of Molecular Variance (AMOVA) after Arlequin v3.5.2.2^79^ on the hierarchical levels showing the highest partition values among groups of species: NW (New World)/OW (Old World) hakes using ITS1 variants; NW hakes (Atlantic/Pacific/Austral) using ITS1 variants; NW hakes and hake morphotypes using ITS1Nes variants. *F*-statistics were used to estimate the proportion of genetic variation found among species (*F*_ST_), among species within groups (*F*_SC_) and among groups (*F*_CT_); *p < 0.001.

Hierarchical groups	Source of variation	d.f	Sum of squares	Variance components	Percentage of variation	Statistics
**NW/OW (ITS1)**						
OW group *M. merluccius* *M. senegalensis* *M. capensis* *M. polli* *M. paradoxus* NW group *M. albidus* *M. bilinearis* *M. gayi* *M. angustimanus* *M. productus* *M. australis* *M. hubbsi*	Among groups	1	428.677	9.007 Va	63.22	*F*_*CT*_ = 0.632*
	Among species within groups	10	326.273	5.091 Vb	35.73	*F*_*SC*_ = 0.357*
	Within species	72	10.728	0.149 Vc	1.05	*F*_*ST*_ = 0.989*
	Total	83	765.679	14.246		
**NW Atlantic**/**Pacific**/**Austral (ITS1)**						
Atlantic group *M. albidus* *M. bilinearis* Pacific group *M. gayi* *M. angustimanus* *M. productus* Austral group *M. hubbsi* *M. australis*	Among groups	2	113.268	3.989 Va	69.95	*F*_*CT*_ = 0.700*
	Among species within groups	4	37.668	1.541 Vb	27.02	*F*_*SC*_ = 0.270*
	Within species	38	6.553	0.172 Vc	3.02	*F*_*ST*_ = 0.970*
	Total	44	157.489	5.703		
**NW hakes and morphotypes (ITS1Nes)**						
Atlantic group *M. albidus* *M. bilinearis* Pacific group *M. gayi* *M. angustimanus* *M. productus* *M. patagonicus* *M. tasmanicus* *M. polylepis* Austral group *M. hubbsi* *M. australis*	Among groups	2	65.727	1.039 Va	58.47	*F*_*CT*_ = 0.892*
	Among species within groups	8	37.409	0.659 Vb	37.07	*F*_*SC*_ = 0.955*
	Within species	85	6.750	0.079 Vc	4.46	*F*_*ST*_ = 0.584*
	Total	95	109.885	1.778		

### Phylogenetic reconstruction

**Figure 2:**
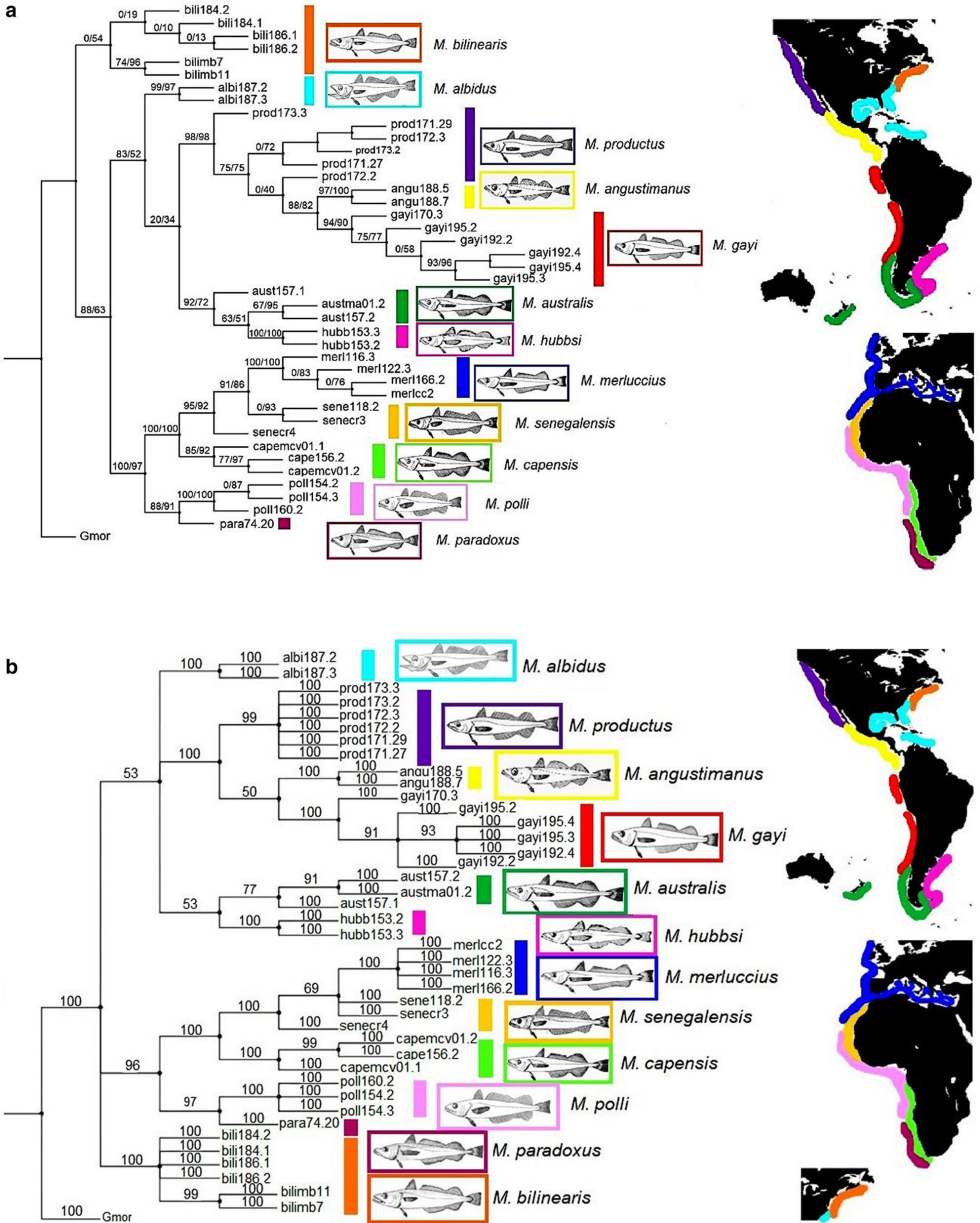
Phylogeny of genus *Merluccius* spp. comprising 11 valid species and *M. angustimanus* as built with concatenated sequences from ITS1-rDNA (HKY85 + I + G substitution model) and cyt b (GTR + G substitution model), (**a**) Maximum likelihood tree (-*lnL* = -4244.841) from IQ-TREE^87^; supporting values are written on branches (SH-aLRT (%)/ultrafast bootstrap (%)). (**b**) Bayesian tree from MRBAYES v3.2.6^86^; branches are annotated with bootstrap values, resp. percent posterior probabilities. OTU Gmor corresponds to the outgroup *Gadus morhua*. Sample codes are given in Table 1 and are followed by the alphanumeric entry code for each specimen in the authors laboratory, e.g. aust_ma01_2 is the specimen No. 2 of sample ma01 from *M. australis* (aust). The maps have been modified after^13^.

Evaluation of the relationship between the best phylogenetic signal and the most plausible phylogenetic scenario upon previous knowledge on this genus showed that Bootstrap support from Bayesian inference was maximum on concatenated ITS1—cyt b sequences (Table 6). The most congruent topology from likelihood-based trees was issued from concatenated ITS1—cyt b sequences (Fig. 2a; Table 6).

**Table 6:** Assessment of evolutionary models, marker types and phylogenetic hypotheses. *NW* New World hakes; *OW* Old World hakes. The bootstrapping values are averaged figures within trees.

DNA data	Bayesian Inference		Maximum likelihood			Common features
	Bootstrap (%)	Tree topology	-*lnL*	Bootstrap (%)	Tree topology	
ITS1 spacer (variants)	85.07	Strong superclade split Absence of polytomy *M. hubbsi* is ancestor to the NW superclade (BEAST, Fig. 1a)	− 1285.51	70.17	Correct superclade split Internal polytomy Unclear ancestral species (PAUP, Supp. Figure S2a)	*M. angustimanus* and *M. productus* share the same haplotype
Cytochrome b gene (Cyt b) (haplotypes)	85.34	Strong superclade split Absence of polytomy *M. bilinearis* is ancestor to the NW superclade (BEAST, Fig. 1b)	− 1137.28	77.00	Poor definition of the OW superclade High impact of polytomy Unclear ancestral species (PAUP, Supp. Figure S3a)	Good phylogenetic signal: species-specific haplotypes (including *M. angustimanus*) and high bootstrap support values
ITS1-Cyt b (concatenated)	86.68	Strong superclade split Medium impact of polytomy *M. bilinearis* is polytomic with both superclades (MRBAYES, Fig. 2b)	− 4244.84	68.00	Strong superclade split Absence of polytomy *M. bilinearis* is ancestor to both super clades (IQ-TREE, Fig. 2a)	Good phylogenetic signal: species-specific haplotypes, high bootstrap values and low impact of polytomy

#### ITS1-based phylogeny

The phylogenetic reconstruction of ML (-*lnL* = 1285.511) performed with PAUP on ITS1 variants recovered a correct supercluster split, a large polytomy within superclusters and an unclear age status of extant species (Supp. Figure S2a). The Bayesian reconstruction from MRBAYES on ITS1 variants recovered a single polytomy comprising all the well-recognized species clusters (Supp. Figure S2b). The Bayesian reconstruction of BEAST on ITS1 variants supported a correct supercluster split, the absence of polytomies and the odd basal placement of *M. hubbsi* in the New World supercluster (Fig. 1a).

#### Cyt b-based phylogeny

Likelihood computation of *dN* and *dS* using HyPhy package^35^ on cyt b sequences showed that six out of 142 codons contained nonsynonymous substitutions. Provided that *p*-values were not significant, the null hypothesis of neutral evolution was accepted. The best –*lnL* value and bootstrap support were observed on cyt b haplotypes but its topological features were unsatisfactory due to multiple polytomies (Supp. Fig S3a; Table 6). The ML phylogenetic reconstruction on cyt b haplotypes (-*lnL* = 1137.282) worked out with PAUP showed a poor definition of the Old World supercluster with a high impact of polytomy (Supp. Figure S3a). The best topology among Bayesian-based supported trees was obtained upon inference on cyt b haplotypes (Fig. 1b). The Bayesian phylogenetic reconstruction from MRBAYES on cyt b haplotypes recovered a strong supercluster split and a polytomy within the New World supercluster to which *M. bilinearis* was ancestor (Supp. Figure S3b). The Bayesian phylogenetic reconstruction of BEAST on cyt b haplotypes recovered a well-supported both, supercluster split and cladogenesis within superclusters, where *M. bilinearis* was basal to New World hakes (Fig. 1b).

#### Concatenated ITS1—cyt b phylogeny

**Figure 3:**
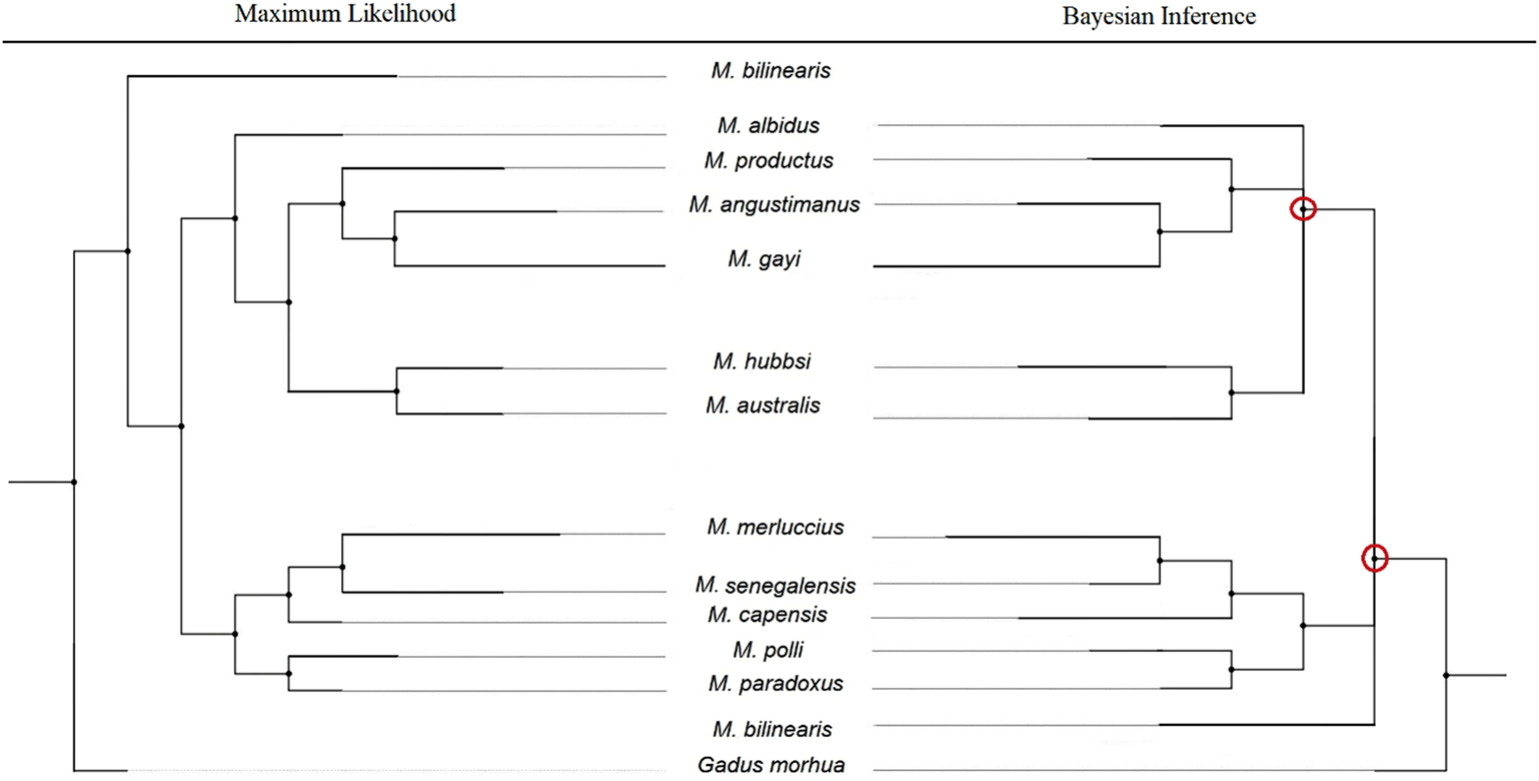
Comparison of reconstruction methods from a synthetic phylogeny of genus *Merluccius* spp. on concatenated sequences from ITS1-rDNA (HKY85 + I + G substitution model) and cyt b (GTR + G substitution model). Left: Phylogeny of genus *Merluccius* spp. using Maximum likelihood (-*lnL* =  − 4244.841) after IQ-TREE^87^. Right: Bayesian tree after MRBAYES v3.2.6^86^. Circled nodes indicate topology differences between both reconstruction methods. *Gadus morhua* was used as outgroup.

The phylogenetic reconstruction using concatenated sequences of both genes showed bootstrap values > 90% for species and clusters. ML (Fig. 2a) and Bayesian (Fig. 2b) methods recovered two superclusters (New World *vs.* Old World), each of which contained two clusters (Pacific North + Pacific South, and North Euro-Africa + South Africa, respectively), the ancient status of *M. bilinearis* to both superclusters and that of *M. albidus* to the New World one. Consistently across reconstructions, *M. angustimanus* branched between *M. productus* and *M. gayi*. The Bayesian reconstruction placed *M. angustimanus* at the base of the sister taxons of *M. productus* and *M. gayi* (Supp. Figure S4). The phylogenies inferred from the methods ML and Bayesian were topologically similar to each other (Fig. 3). The Bayesian reconstruction failed to resolve the phylogenetic status of *M. bilinearis* regarding the two superclusters and exhibited a large polytomy within the New World supercluster.

#### Coalescence

**Figure 4:**
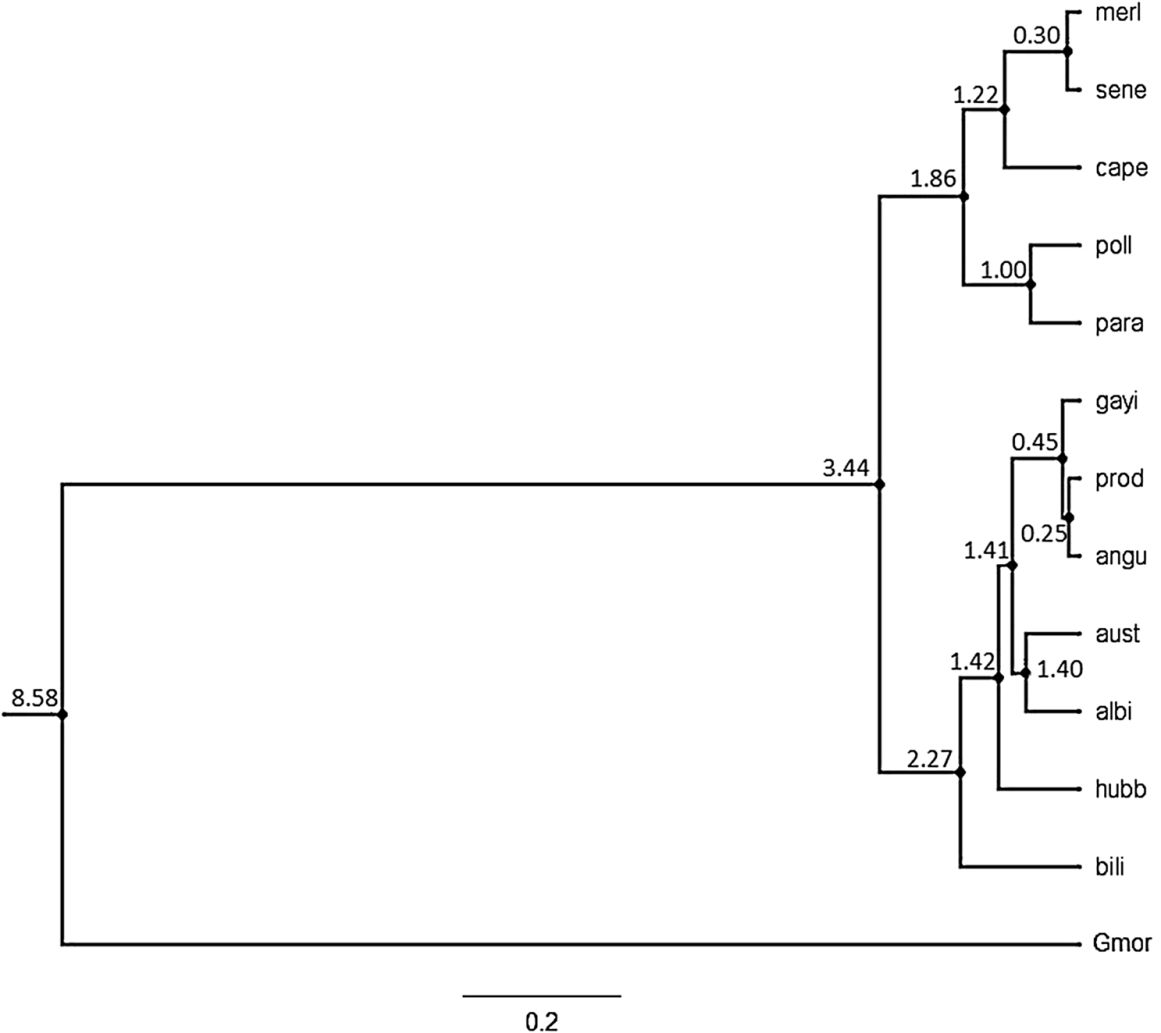
Bayesian reconstruction of genus *Merluccius* spp. on sequence variation of cyt b majority haplotypes (GTR + G substitution model) using BEAST v1.8.4^85^. Divergence time is given in MYA on nodes as calculated upon a 2% mutation rate averaged among 26 pairs of major intraspecific phylogroups of fishes^40^. Gmor corresponds to the outgroup *Gadus morhua*. Scale bar indicates the No. of nucleotide substitutions per 100 DNA residues. Sample codes are given in Table 1.

The relative coalescence times inferred from a typical 2% mutation rate of cyt b in fish was ~ 9 MYA between *Merluccius* and the codfish genus *Gadus,* ~ 3.5 MYA between the New World and the Old World superclusters, and ~ 2.3 MYA between *M. bilinearis* and other New World hakes (Fig. 4). Atlantic and Pacific New World hakes would have diverged some ~ 1.4 MYA.

#### ITS1Nes-based phylodiagnosis

**Figure 5:**
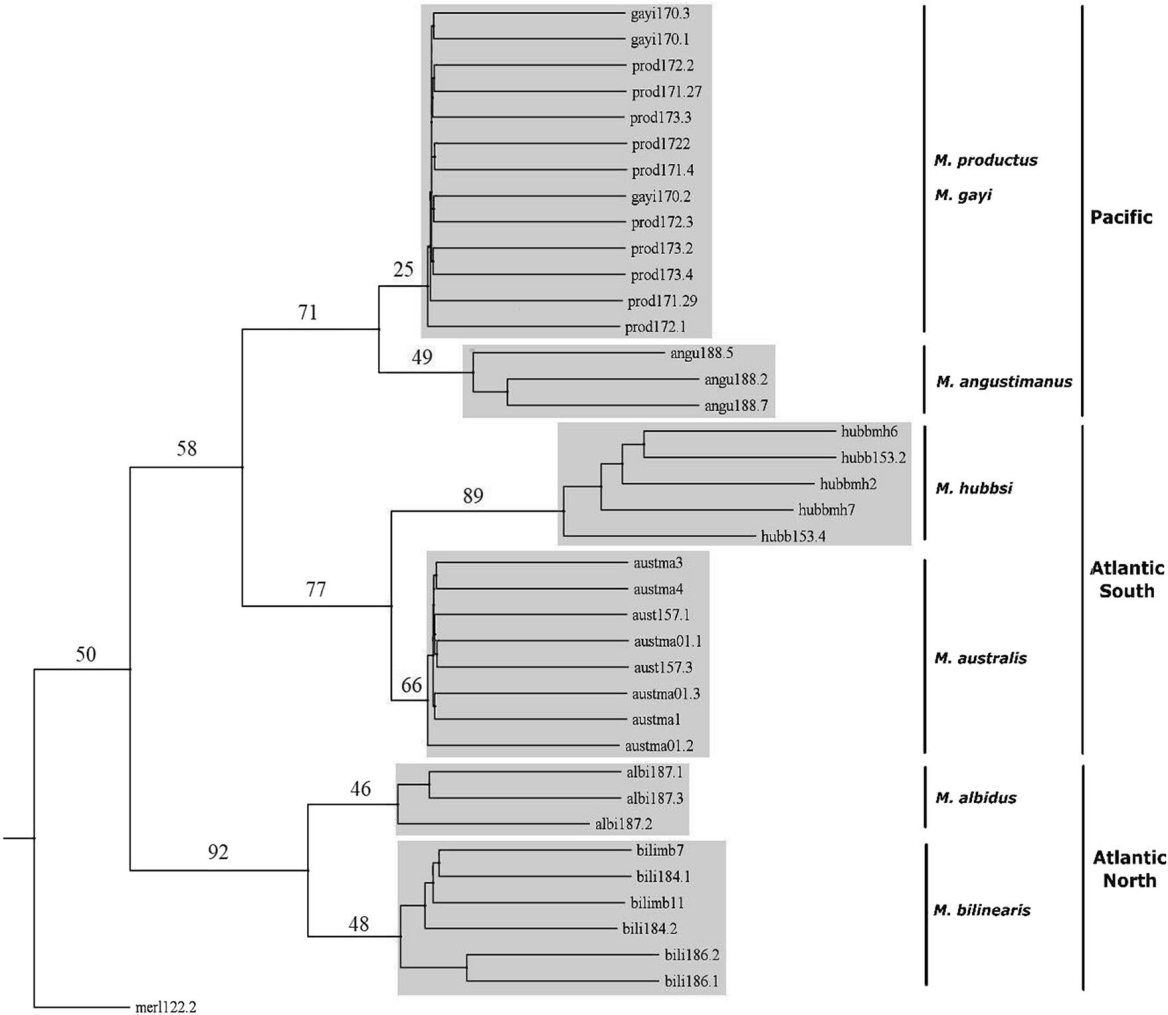
Basal NJ tree on ITS1Nes sequences after PHYLIPv3.6^84^ built to reconstruct the phylogenetic backbone of genus *Merluccius* spp. from the New World. The European hake (merl) was used as outgroup. Gray rectangles delineate a single cluster for each valid species. The percentage of trees over 1000 bootstrap replicates are shown above branches. Codes for valid species are given in Table 1 and are followed by the alphanumeric entry code for each specimen in the authors laboratory, e.g. aust_ma01_3 is the specimen No. 3 of sample ma01 from *M. australis* (aust).

**Figure 6:**
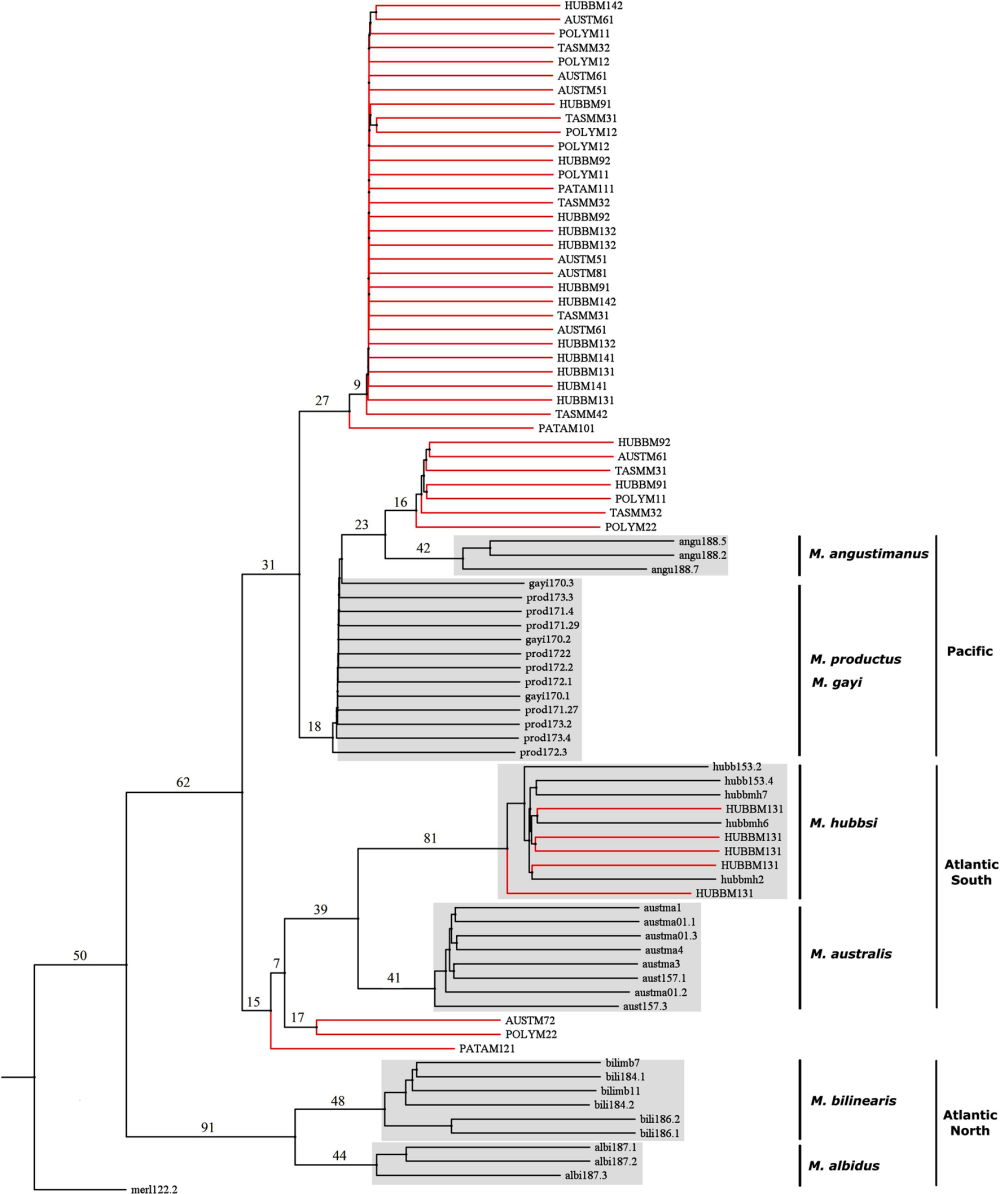
Phylodiagnostic NJ tree (sensu^18^) on ITS1Nes sequences after PHYLIPv3.6^84^ built to allocate morphotype clones (red branches and codes in uppercase) to the phylogenetic backbone of genus *Merluccius* spp. from the New World (clusters in grey rectangles, black branches and codes in lowercase). The percentage of trees over 1000 bootstrap replicates are shown above branches. Codes for valid species are given in Table 1 and are followed by the alphanumeric entry code for each specimen in the authors laboratory, e.g. aust_ma01_3 is the specimen No. 3 of sample ma01 from *M. australis* (aust). Codes for morphotypes are given in Table 2 and are followed by an alphanumeric entry from the authors laboratories (Supp. Table S9), e.g. TASM_M3_1 is a ITS1Nes sequence from muscle tissue (1) of specimen M3 (M3) of morphotype *M. tasmanicus* (TASM).

The Neighbor-Joining reconstruction performed with the ITS1Nes fragment included six valid species and *M. angustimanus*, and comformed the basal phylogenetic backbone of this genus in the New World against which new phylogenetic hypotheses can be tested. The basal support afforded from the ITS1Nes fragment was quite close to that obtained on the full ITS1 sequence (Fig. 1a) but including a polytomy for *M. gayi–M. productus* (Fig. 5). Re-runs of morphotypes against the basal tree assigned five morphotype clones of *M. hubbsi* to the valid *M. hubbsi* cluster. The remaining 41 morphotype clones (*M. polylepis, M. tasmanicus, M. patagonicus*, *M. australis, M. hubbsi*) were grouped in a large intermediate and weakly supported cluster placed between the Pacific cluster (*M. gayi–M. productus–M. angustimanus*) and the Austral cluster (*M. australis–M. hubbsi*) (Fig. 6). Two additional minor clusters of morphotypes closely branched either to the Pacific cluster or to the Austral cluster.

## Discussion

Genetic homogeneity is the null hypothesis in widely-distributed marine taxa with large population size and sometimes morphological divergence may be a first clue to hypothesize on the existence of genetically divergent units^13^. Demonstration of intraspecific genetic differentiation requires comprehensive spatio-temporal sample designs, the identification of suitable genetic markers at the resolution level concerned, and the choice of appropriate phylogenetic algorithms^18^. The nuclear ITS1-rDNA region has been successfully applied in the identification of closely related taxa^36^, in fish phylogeography^37^, in phylogenetic inference^38^ as well as in forensic authentication of species^33^. Also, the cyt b gene is a well-known mtDNA gene in structure and function^39^ and is useful in phylogenetic reconstruction at many taxonomic levels, including congeneric species and confamiliar genera^40^ but see exceptions^41^. The synergy afforded from concatenated analyses of the nuclear ITS1-rDNA and the mitochondrial DNA cyt b allows comparison of interspecific levels of divergence and to achieving phylogenetic scenarios unaffordable from single-markers approaches^42^. The higher interspecific variation observed between New World and Old World superclusters (e.g. ITS1, *D*_xy_ ≈ 0.090) than within superclusters (e.g. ITS1, *D*_xy_ ≈ 0.040) is expected if previous calibrations were robust^33^. In consequence a higher net evolutionary divergence (*d*) existed among Old World species (*d* ≈ 0.080) than among New World species (*d* ≈ 0.030). In contrast, the minimum interspecific divergences (*d* and *D*_xy_) observed among Pacific New World hakes (*M. gayi–M. productus–M. angustimanus*) represented the most recent evolutionary scenario in this genus, as reported from morphometric data^5^, allozyme data^43^ and mtDNA plus microsatellite data^27^.

The PCoA performed on ITS1 sequences agreed with AMOVA partitions that separated New World hakes into Atlantic, Austral, and Pacific groups. Those exploratory analyses also allocated morphotypes to either the Pacific or the Austral groups. The phylogenetic support for those groups tested between methods (Bayesian inference *vs*. ML) and markers (ITS1, cyt b) with non-parametric bootstrapping produced the best-supported Bayesian-based reconstruction on cyt b haplotypes. Such reconstruction was characterized by a well-defined supercluster split and the placement of *M. bilinearis* at the base of the New World supercluster (BEAST, Fig. 1b). However, the best Bayesian tree and the best ML tree were not fully congruent with previous studies, a handicap if congruence among marker reconstructions is a relevant asset, e.g. the better ML-value on cyt b does not grant any better species tree than that from ML-ITS1 because the topology of the latter is more congruent with previous studies. Such scenario highlights the insufficiency of the ML method on cyt b variation as compared to the Bayesian approach (but see^44^). The Bayesian phylogeny performed on both genes with BEAST was the unique method recovering both, a well-supported supercluster split and a full intracluster resolution, including the full definition of the Austral cluster. Discrepancies between markers using BEAST consisted on both, the branching of *M. hubbsi* as the oldest taxon among New World hakes with ITS1 and the branching of *M. albidus* within the Austral cluster with cyt b. Nonetheless, caution is needed on rejecting those odd positions regarding previous studies, since they could result from differential evolutionary rates among the markers applied.

It is expected that deep phylogenetic rooting of ancient hake lineages could be better afforded from conservative mtDNA haplotypes^45^. However, more recent evolutionary processes such as hybridization and drift could be better unveiled by highly recombinant nuclear DNA markers. Therefore, speciation histories based on mtDNA alone can be extensively misleading and large phylogenetic discrepancies have been reported between nuclear DNA and mitochondrial DNA^46^. Successful reconstructions have been achieved on concatenated sequences of the ITS region and COI^47^ or on nuclear genes^48^. Also, current data showed that concatenated data from ITS1*-*rDNA and cyt b at reconstructing hakes phylogeny using Bayesian inference have dramatically improved topology and support regarding individual markers^49^. Letting alone the well-supported supercluster split and the absence of polytomy, three topological novelties consisted on the placement of *M. bilinearis* as the oldest species of the genus, the basal branching of *M. albidus* to all New World hakes, and the intermediate branching of *M. angustimanus* between *M. gayi* and *M. productus* (see IQ-TREE, Fig. 2a). Those unexpected scenarios in view of previous studies can be explained as a synergical advantage of data concatenation as genes add up to produce a more balanced signal of the interspecific evolutionary divergence than that afforded from single genes^47^. Since each gene responds to distinct evolutionary dynamics it is expected that concatenated gene reconstructions approach the average evolutionary signal of their common history^50^.

The major phylogenetic split in *Merluccius* comprises two monophyletic superclusters, the Old World one comprises five species and the New World one comprises six species, as shown with parasites^5,8,51^, morphology and meristic traits^6^, allozymes^14,52^ and nucDNA^33^. This genus is believed to emerge in the Cretaceous after the opening of the southern Atlantic Ocean basin between South America and Africa^7^. However, its evolutionary bifurcation into New World and Old World superclusters is believed to have begun in the Oligocene^53^ when the northern margins of those continents begun to diverge (~ 30 MYA^54^). Application of an average 2% evolutionary clock to the cyt b as calibrated on 23 fishes^40,55^ suggests that genus *Merluccius* and genus *Gadus* diverged some 9 MYA and the supercluster split would have taken place some 3.4 MYA, what is congruent with estimates from the evolutionary rate of four mtDNA genes^56^ which dated back the supercluster split to some 3.0–4.2 MYA^57^. Such major evolutionary split seems to have been determined by a rapid vicariant-subsequent speciation in both continents in the early Pleistocene (*ca*. 2.58–0.77 MYA) with separation of Old World hakes into two clusters around 1.9 MYA, i.e. much later than reported after allozyme data (4.2–3 MYA^52^). The actual speciation scenario was not accomplished until ~ 0.3 MYA with the divergence within the species pairs *M. merluccius–M. senegalensis* and *M. productus–M. gayi*. Nevertheles, such divergence times as computed on a cyt b—based molecular clock should be taken as relative inferences because the evolutionary scenario from concatenated-based trees differs substantially from any other single-marker reconstruction. The Old World supercluster is believed to have experienced strong bottlenecks after the early divergence of *M. polli* and *M. paradoxus,* followed by a later speciation of *M. merluccius*, *M. capensis* and *M. senegalensis*^52^. A consensus exists on the monophyly of those two clusters^8,14,15,52^. Such phylogenetic partition is biogeographically counterintuitive since sympatric *M. capensis* and *M. paradoxus* in Atlantic South Africa are believed to emerge from two independent dispersions of North Atlantic taxa along the west coast of Africa^58^. Moreover, provided that *M. polli* and *M. paradoxus* are two benthonic species closer to the New World supercluster, they have been proposed both, either to descend from an early Old World *Merluccius* cluster^52^ or to represent a distinct speciation process in the Eastern Atlantic^59^.

The variance partition, the multivariate approach, and the phylogenetic inference, all them support three clusters within the New World supercluster, namely the Atlantic, the Austral and the Pacific. In the Atlantic cluster, the evolutionary status of *M. bilinearis* is disaggregated in classical hake phylogenies. Generally, this species has been placed as ancient to New World species with various methods and markers, including likelihood^52^ and parsimony^14^ on allozymes; NJ, ML, UPGMA on the D-loop^15^ and current Bayesian analyses on cyt b haplotypes. However, new scenarios appear after concatenated ITS1—cyt b sequences which indefectibly place *M. bilinearis* as the oldest among extant hake species (see Fig. 2)**.**

Most previous studies agree on that hake originated in the North Atlantic and entered the Pacific, but disagree on the origin of *M. hubbsi*, i.e. as diverted either from an eastern south Pacific stock^6,60^ or from a western north Atlantic stock^5,8^. A novelty in the current concatenated approach is a well-supported Austral cluster that is congruent with parasite studies in which *M. hubbsi* and *M. australis* are closely related taxa of Austral origin^6^. The concatenated ML tree also shows the basal status of *M. albidus* to all New World hakes except *M. bilinearis*, what agrees with previous morphological and meristic studies^5^. Moreover, its broad western Atlantic distribution suggests that *M. albidus* is the colonizer of de Southern Cone and the primeval species of the Austral cluster. The phylogenetic proximity of *M. hubbsi* and *M. australis* suggests that *M. hubbsi* would have speciated after *M. albidus* some 1.5 MYA and would have reached its present distribution by a dispersal route along the Atlantic coast^5^. Noteworthy, other marker types offer the different scenario of *M. hubbsi* speciation prior to *M. albidus* (see also^14^).

Pacific New World hakes are believed originated upon the early migration of North Atlantic New World hakes into the Pacific^8^. Combined allozyme data and mtDNA^14,52^ and hake otoliths^61,62^ support the hypothesis of the separation between *M. bilinearis* and both*, M. productus–M. gayi* and *M. albidus* before the closure of the Panama Isthmus (3.5 MYA^63^) and advanced the divergence of *M. bilinearis* to the Miocene some 12 MYA^52^. However, present data suggest that *M. albidus* diverged first from *M. bilinearis* and that the divergence of actual north Pacific species from Atlantic species begun with the rise of the Panama Isthmus. A more recent speciation in the Pacific seems to have occurred between *M. productus* and *M. gayi* some 0.5 MYA. Such temporal estimate is congruent with the origin of *M. angustimanus* as a result of a post-speciation confinement of one of those species or their hybrids in the northern Gulf of California, some 0.25 MYA. Those hypothetical timeframes should be further investigated in relation to reported population fragmentations and expansions due to climatic oscillations that took place during Pleistocene glaciations in that area^12^.

**S**everal species have been proposed to occur in the Pacific New World cluster such as *M. productus, M. angustimanus, M. hernandezi*, *M. gayi gayi,* and *M. gayi peruanus*^22,64,65^ as well as morphs (dwarf, normal, etc.)^27,66,67^ all with close morphological and meristic similarity among each other^5,6^ as well as in protein-coding loci^43^. Recently, it has been suggested that *M. gayi*^68^ could be the single pan-Pacific species^28^ although the North-Eastern Pacific hake has to be named *Merluccius productus* by priority^28^. Despite being considered by FAO as a variant of *M. angustimanus*^3^ and reproductively isolated from *M. productus* and *M. angustimanus*^14^, *Merluccius hernandezi*^22^ from Sinaloa state would likely be a dwarf morphotype of *M. productus*^28^. After its first description as a “*tropical deep-water species off the Pacific coast of Central America from Mexico to Panama which is sandwiched between M. productus to the north and M. gayi to the south*”^14^, *M. angustimanus* has been a more recognized taxon than *M. hernandezi*^69^*.* In the first genetic description of *M. angustimanus*^33^ it was named *M. hernandezi* provided its northern California origin. Following recovering of its trawling data from the seventies^70^ as well as the examination of its large scales (Mathews, personal communication) such sample was properly renamed as *M. angustimanus*^18^. That genetic analysis of *M. angustimanus* showed that the PCR–RFLP pattern on the ITS1 spacer was very similar to those of *M. productus* and *M. gayi*^18,33^. Also, current data showed that *M. angustimanus* shared its two unique ITS1 variants with *M. productus.* That result is congruent with the within species concerted evolution of the ITS1-rDNA gene family^37^ and also with the recently proposed confinement and drift of *M. productus* in the Gulf of California giving rise to its present divergent population^27,27^. Present data also suggest the putative hybrid origin of *M. angustimanus* by means of a trans-equatorial incursion of *M. gayi*^5,6,14,22^ in the territories of *M. productus* in the Gulf of California (see the bootstrap weakness within the Pacific cluster in all concatenated reconstructions, Fig. 2). Moreover, the six specimens of *M. angustimanus* examined had no evolutionary novelties in their cyt b region but rather were a chimeric genome from extant neighboring species plus an A-139 residue shared with *M. albidus* (see Supp. Table S4). In summary, *M. angustimanus* seems to be a hake population trapped in the northern Gulf of California^27^ whose origin dates back some 0.25 MYA either from hybridization between *M. productus* and *M. gayi* or from a confinement of a subset of those species therein. Such hypotheses would explain the weak morphological and molecular differentiation of *M. angustimanus* from its neighboring hake species^22^. Whether that confined population can be self-sustainable as a relic of an ancient hybridization or if its viability depends on ongoing genetic contribution from the surrounding *M. productus*, are unexplored questions.

The morphotypes under test other than *M. angustimanus* were analyzed with the nested fragment ITS1Nes which offered a more conservative view of the interspecific diversity than the full ITS1 sequence, i.e. 11 *Hake*ITS1Nes variants *versus* 19 *Hake*ITS1 variants. In addition to morphotype-specific variants, all morphotypes shared the specific HakeITS1Nes.2 sequence from Pacific hakes, what suggests their common origin. Such commonality does not preclude the presence of specific sequences within the ITS1 spacer family that trace back the origin of their carriers. For instance, although some ITS1Nes variants of a given morphotype fully grouped into the *M. hubbsi* clade (e.g. HUBBM131) other intraindividual variants branched intermediately between the Pacific cluster and the Austral cluster. That is a typical scenario for interspecific hybrids which can be ascertained by inspection of the dramatic decrease of the basal tree bootstrap support of the Austral and the Pacific clusters in the phylogenetic backbone (see Figs. 5 and 6). The significant No. of recombination events detected in those sequences also comes to support the working hypothesis of a hybrid origin for those morphotypes. The small molecular differentiation observed among morphotypes does not support either their distinct origin nor the existence of cryptic species around the Southern Cone. For instance, the six clones of the *M. hubbsi* paratype from the Beagle Channel (HUBBM91 and HUBBM92) shared ITS1Nes variants with all other morphotypes, including *M. patagonicus* (PATAM101 and PATAM111). After the description of the *M. patagonicus*^23^ some additional analyses suggested its synonymy with *M. hubbsi*^29,31^. However, the three paratypes of *M. patagonicus* did not share any sequence variant with true specimens of *M. hubbsi* (e.g. hubb153.2) but branched between the clusters of Austral hakes and Pacific hakes. Likewise, *M. tasmanicus*^24^ and *M. polylepis* from the Southern Ocean^32^ had been proposed as synonymous of *M. australis*^6,29,30^. However, their phylogenetic status was much like the rest of morphotypes, e.g. the paratypes of *M. australis* from Chile and from New Zealand. It is probable that those rare morphotypes resemble extant species when analyzed with more conservative markers such as the mtDNA *Cytochrome c* oxidase subunit I (COI)^29^. However, less conservative regions such as the ITS1-rDNA suggest that the morphotypes examined are the result of recurrent hybridization between adjacent *M. gayi* and *M. australis* or *M. hubbsi* in the Southern Cone. The wide distribution range of those parent species would make probable to find hybrids commonplace from the western Atlantic South in Argentine to the Western Pacific South in New Zealand.

## Methods

### Sample collection

This study was conducted in accordance with the European directive 2010/63/EU and the Spanish legislation on animal welfare (RD53/2013 and RD1386/2018). All the experiments were approved by the local ethics committee of Centro Oceanográfico de Vigo. Samples were taken from fishing or formalin-preserved corps; therefore no restrictions apply on taking tissue samples for species identification. Eleven valid hake species were sampled in their natural ranges (Table 1) together with *M. angustimanus* from the northern Gulf of California and nine morphotypes as three from the western Atlantic South, three from the eastern Pacific South and three from the south Western Pacific (Table 2, Supp. Table S9). The Atlantic cod (*Gadus morhua*) was used as a phylogenetic outgroup. The world-wide sampling was performed on board of factory ships and research vessels between 2000 and 2003^33^ when 9–277 specimens per species were preserved in 95% ethanol or frozen upon collection and their GPS recorded on board (Table 1). All specimens were identified using species-specific morphological traits such as otoliths, shape of abdominal vertebrae (parapophysis), cranial shape and pectoral fin length, following classification criteria previously established^6^. Whole specimens were boiled to recover those structures and to facilitate bone cleaning. Six specimens of *M. angustimanus* were sampled as preserved frozen from a bottom trawl campaign (80–523 m in depth) carried out in 1973 by Instituto Nacional de la Pesca, Mexico^70^. Fourteen additional specimens as holotypes, paratypes and rare morphotypes were either sampled in situ by the authors or taken from museum specimens as preserved in formalin. Two specimens of *M. tasmanicus* (DM5566 and DM3963) and four specimens of *M. patagonicus* were sampled and described in^23,24^, respectively. The paratype USNM 157765 of *M. polylepis* from Puerto Montt (Chile) was described by^23^ and the *M. polylepis* holotype off Chiloe (Chilean Pacific) was described by^32^. Three rare morphotypes of *M. hubbsi* from the Beagle Channel (IIPB92/1987) and from Puerto Madryn were described by^23^. Finally, two rare morphotypes of *M. australis* from New Zealand (MOVI 27492–27493, formerly NMNZ P.13122) were described by^24^ and two additional ones from the Chilean Pacific (off the Aysén Region) were described in^23^.

### Molecular data

Genomic DNA was extracted with FENOSALT^71^ including a preliminary 24 h hydration step for formalin-fixed samples. Two synergic tools for hakes identification were applied, one based on the ITS1-rDNA spacer^33^ and another based on the mtDNA cyt b gene^18^. Those targets are suitable DNA regions for the taxonomic classification of closely related taxa and their combinatory power has proven to be a robust approach to reduce the authentication error from hake-based commercial products^72^. Amplification conditions for the ITS1-rDNA spacer followed previous developments in this genus^33,73^. Electropherograms were revised with Chromas software (Technelysium, Tewantin, Australia). A total of 85 specimens were sequenced as averaging ~ 6 specimens per species (Supp. Table S1). Formalin-preserved samples produced 46 ITS1 nested sequences (ITS1Nes) of 66 bp in length using the new primer pair, *PARIB152* (5′-GTTTCGCTGACCCCGTTGG-3′) and *PARIB197* (5′-CCGCACTCTCCCTCGTACCTC-3′). The PCR reactions contained 1 µl DNA template, 20 pmol of each primer, 200 µM of each dNTP, 1.6 mM MgCl_2_, 5 µl of 5X Colorless GoTaq Flexi Buffer and 1.5U GoTaq Flexi DNA Polymerase (Promega) in a total volume of 25µL. Amplification was performed in an Eppendorf Mastercycler Gradient under the following conditions: one cycle at 95ºC for 5 min, 30 cycles of 1 min at 95 °C, 1 min at 57 °C and 1 min at 72 °C and a final extension at 72 °C for 10 min. Amplicons were cleaned using the Wizard SV Gel and PCR Clean-Up System (Promega) according to the manufacturer’s protocol. Clean PCR products were cloned into the pGEM-T Easy Vector System II (Promega) following the manufacturer protocol using a 3:1 insert:vector ratio. Clones were lysed and their plasmid purified using the NucleoSpin Extract (Mackerey -Nagel). Sequencing was performed on both strands in an ABI Prism 3100 Sequencer (Applied Biosystems) using T7 and SP6 primers. Several ITS1Nes sequences were obtained from multiple cloning per specimen of each morphotype (Supp. Table S1, Supp. Table S9). The 3′-end of the cyt b gene was obtained as described for this genus^18,74^. Sixty-six specimens were sequenced for cyt b as 5.42 ± 2.47 specimens per species in addition to the outgroup *Gadus morhua*.

### Sequence alignment

The 3′ end of the 18S gene and the 5′-end of the 5.8S gene were used to align the 85 ITS1 sequences obtained from 11 hake species using the program SeqLab from the GCG software package (Genetics Computer Group, Madison, Wisconsin)^75^. The alignment of 66 sequences from the 464–465 bp cyt b region was performed from the 3′-end of the mitochondrial tRNA-*Glu* gene. The molecular properties of those sequences per species were calculated using MEGA v7.0.20^76^. Nucleotide diversity (*P*_*i*_) was computed using DnaSP v5.10.1^77^. The average number of nucleotide substitutions per site (*D*_*xy*_) and estimates of the Net Evolutionary Divergence between species (*d*) were compared among taxa using the confidence interval calculated as *CI* = $\stackrel{-}{x}$±1.96*(S.D./$\sqrt{n}$). The Principal Coordinates Analysis (PCoA) built with GenAlEx v6.503^78^ on the molecular variation of ITS1 variants was used to check for the correct divergence signal between superclusters (New World and Old World) as well as between clusters and between species. Genetic variation within and between clusters were contrasted with an Analysis of Molecular Variance (AMOVA) as implemented in Arlequin v3.5.2.2^79^.

### Comparative phylogeny and synthetic phylogeny

The phylogenetic reconstructions using the ITS1 region, the ITS1Nes fragment and the cyt b region included reference samples used at establishing the phylogenetic backbone of the genus^18,33^. The phylogenetic test enforced to allocate morphotypes was based on the phylogenetic backbone of New World hakes built with the ITS1Nes fragment. The most suitable nucleotide substitution models were selected using jModeltest v2.1.10^80^. The HKY85 + I + G model^81^ was the best fitted to the ITS1-rDNA variation and the GTR + G model^82^ was the best fitted to that of the cyt b gene. Likelihood computations of nonsynonymous (*dN)* and synonymous (*dS)* substitution rates were conducted using HyPhy software package^35^. The phylogenetic reconstruction was carried out using full sequences (ITS1*,* ITS1Nes and cyt b) as well as sequence variants (ITS1 and ITS1Nes) and cyt b haplotypes, as obtained with program DnaSP v5.10.1^77^. That software was also used to estimate the Recombination parameter (*R*)^34^ as (*R* = *4Nr*) where *N* is the population size and *r* is the recombination rate per sequence. The rate R allowed to assess whether recombination fingerprints can be detected among ITS1Nes sequences as expected in highly recombinant nuclear DNA markers^37^.

Character-based phylogenies were first constructed using Maximum Likelihood (ML) hypothesis testing after PAUP v4.0^83^, a gamma distribution coefficient (gamma-ITS1 or gamma-ITS1Nes = 0.269; gamma—cyt b = 0.420), a transition/transversion rate (*T*_*i*_*/T*_*v*_*-*ITS1 and *T*_*i*_*/T*_*v*_*-*ITS1Nes = 1.761; *T*_*i*_*/T*_*v*_*—*cyt b = 7.143) and the nucleotide substitution rate matrix observed for each marker. An algorithmic Neighbor-joining tree was built on ITS1Nes using PHYLIP v3.6 upon 5000 bootstrap replicates^84^. Bayesian phylogenetic hypotheses on ITS1 variants as well as on cyt b haplotypes were tested using the programs BEAST v1.8.4^85^ and MRBAYES v3.2.6^86^ and taking into account the best fitted evolutionary models. The comparative phylogeny consisted on contrasting tree support values and topological consistence per marker type (ITS1 and cyt b) among reconstruction methods (ML and Bayesian). The synthetic phylogeny of the genus consisted on a ML reconstruction made upon concatenated data sets of both genes using IQ-TREE (http://iqtree.cibiv.univie.ac.at/^87^) which allows partitioning the analysis to simultaneously conjugate two evolutionary models and also enforces the Shimodaira–Hasegawa test (SH) for testing gene trees. The null hypothesis (H_0_) stated that all the trees tested would be equally performant at explaining the observed data, while the alternate hypothesis (H_1_) stated that only one among several trees was a better proxy to real data. The Bayesian models implemented in MRBAYES and BEAST were employed in the phylogenetic reconstruction of concatenated sequences. The Net Evolutionary Divergence (*d*) among sequences (ITS1 variants, ITS1Nes variants, cyt b haplotypes) was calculated upon divergence between OTUs (species, clusters and superclusters) as (*P*_AB_) and corrected for within-OTU variation (*P*_A_ and *P*_B_) as:

$$P_{AB} \left( {net} \right) = P_{AB} - 0.5\left( {P_{A} + P_{B} } \right)$$

The net divergence values among cyt b sequences were converted to proxy estimates of coalescence times using a standard mtDNA clock for fishes, i.e. 2% sequence divergence per million years between two lineages^55^.

## Supplementary Information

Supplementary Information 1.

Supplementary Information 2.
