# Supplementary material for: Phylogenetic prospecting for cryptic species of the genus Merluccius (Actinopterygii: Merlucciidae)
Source: Sci Rep. 2021 Mar 15;11:5929. doi: 10.1038/s41598-021-85008-9 (PMC7961003; doi:10.1038/s41598-021-85008-9)
Supplement: Supplementary file 1 — Supplementary Information 1. [file 41598_2021_85008_MOESM1_ESM.docx]

**Phylogenetic prospecting for cryptic species of the genus Merluccius (Actinopterygii: Merlucciidae)**

**Montse Pérez^1^, María Fernández-Míguez^1,2^, Jesús Matallanas^3^, Domingo Lloris^4^ and Pablo Presa^2,*^**

^1^AquaCOV, Centro Oceanográfico de Vigo, Instituto Español de Oceanografía, 36390 Vigo, Spain.

^2^CIM-Universidad de Vigo, Laboratorio de Recursos Genéticos Marinos, Facultad de Biología, 36310 Vigo, Spain.

^3^Unidad de Zoología, Departamento de Biología Animal, Biología Vegetal y Ecología, Universidad Autónoma de Barcelona, 08193, Spain.

^4^Institut de Ciències del Mar (CMIMA-CSIC), Barcelona, 08003, Spain

*[pressa@uvigo.es](mailto:pressa@uvigo.es)


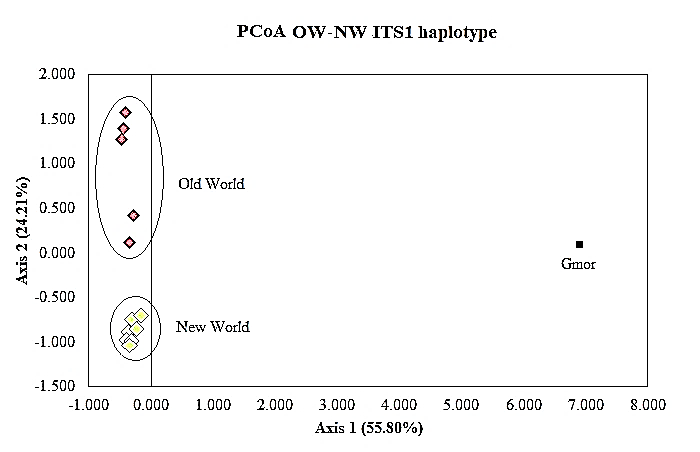
**a**

**
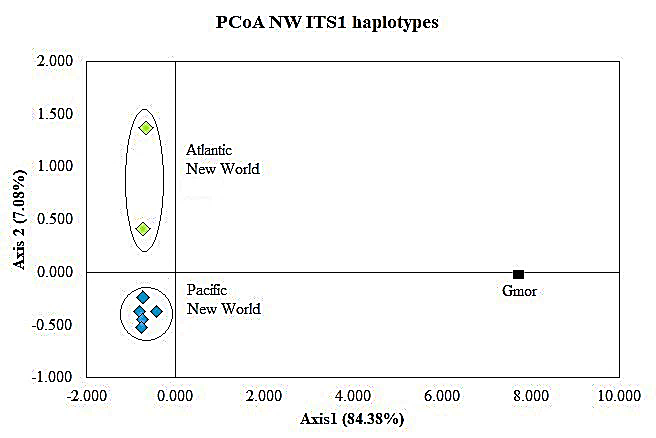
b**


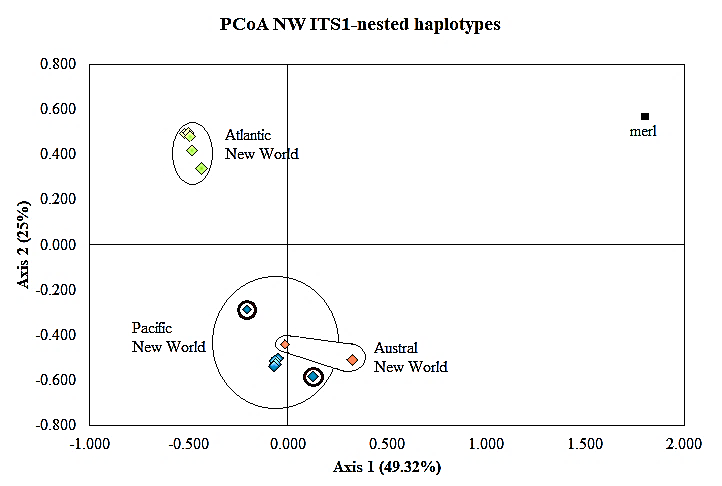
**c**

**Supplementary Figure S1.** Principal Coordinates Analysis (PCoA) built after the molecular variation of ITS1 variants using GenAlEx v6.503 ^[79]^; **a)** Old World (OW) hakes (orange symbols: *M. merluccius, M. senegalensis, M. capensis, M. polli* and *M. paradoxus*), New World (NW) hakes (yellow symbols: *M. productus, M. gayi, M. angustimanus, M. australis, M. albidus, M. hubbsi* and *M. bilinearis*), and Atlantic cod (black symbol, *Gadus morhua*); **b)** Atlantic NW hakes (green symbols: *M. albidus* and *M. bilinearis*), Pacific NW hakes (blue symbols: *M. australis, M. angustimanus, M. productus* and *M. gayi),* and Atlantic cod (black symbol, *Gadus morhua*); **c)** PCoA built after the molecular variation of ITS1Nes variants from Atlantic NW hakes (green symbols, *M. albidus, M. bilinearis*), Pacific NW hakes (blue symbols: *M. angustimanus, M. productus, M. gayi*), Austral NW hakes (orange symbols, *M. hubbsi* and *M. australis*) and morphotypes (red circled blue symbols: *M. tasmanicus*, *M. patagonicus* and *M. polylepis*). The European hake (merl, *Merluccius merluccius*) is the outgroup.

**
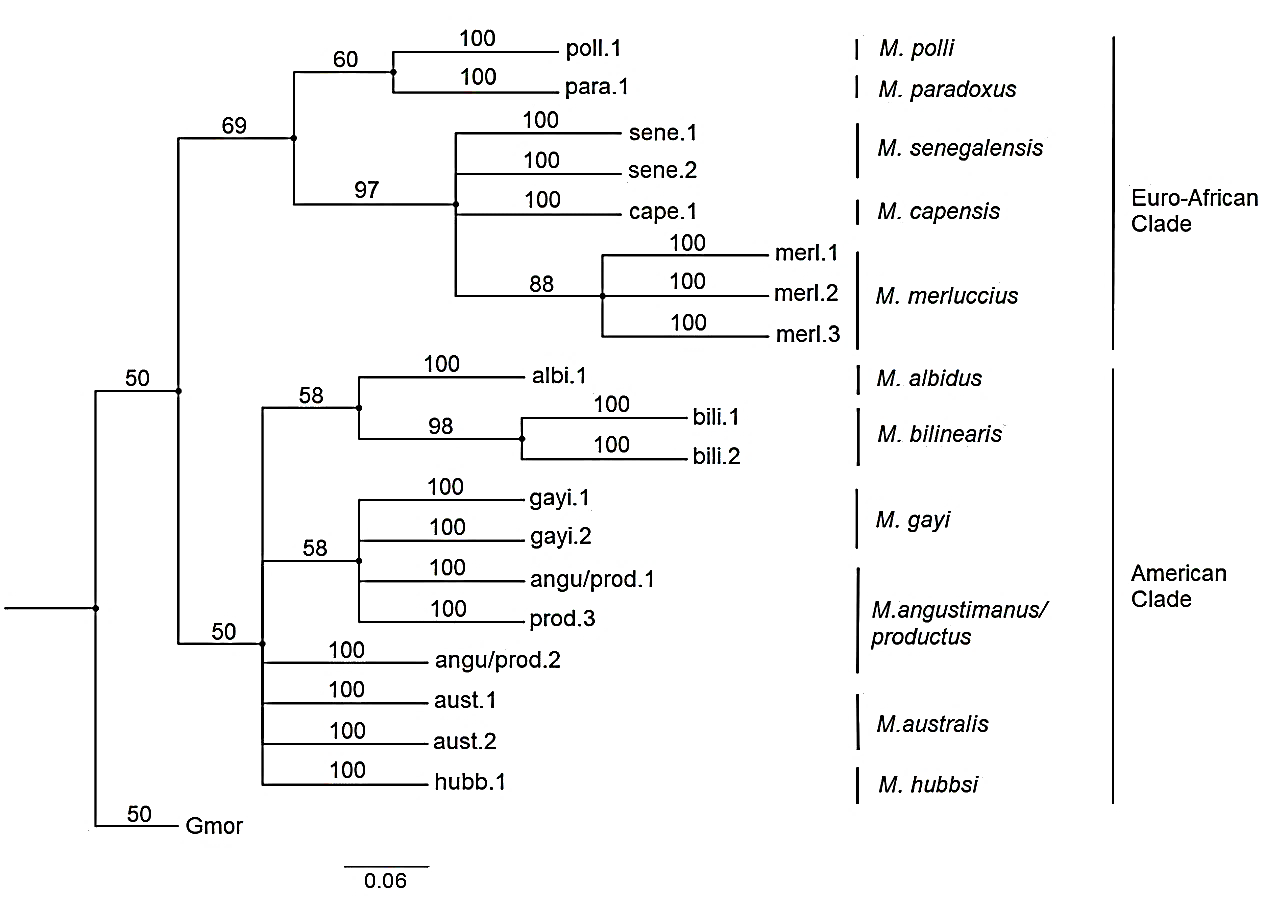
a**

**
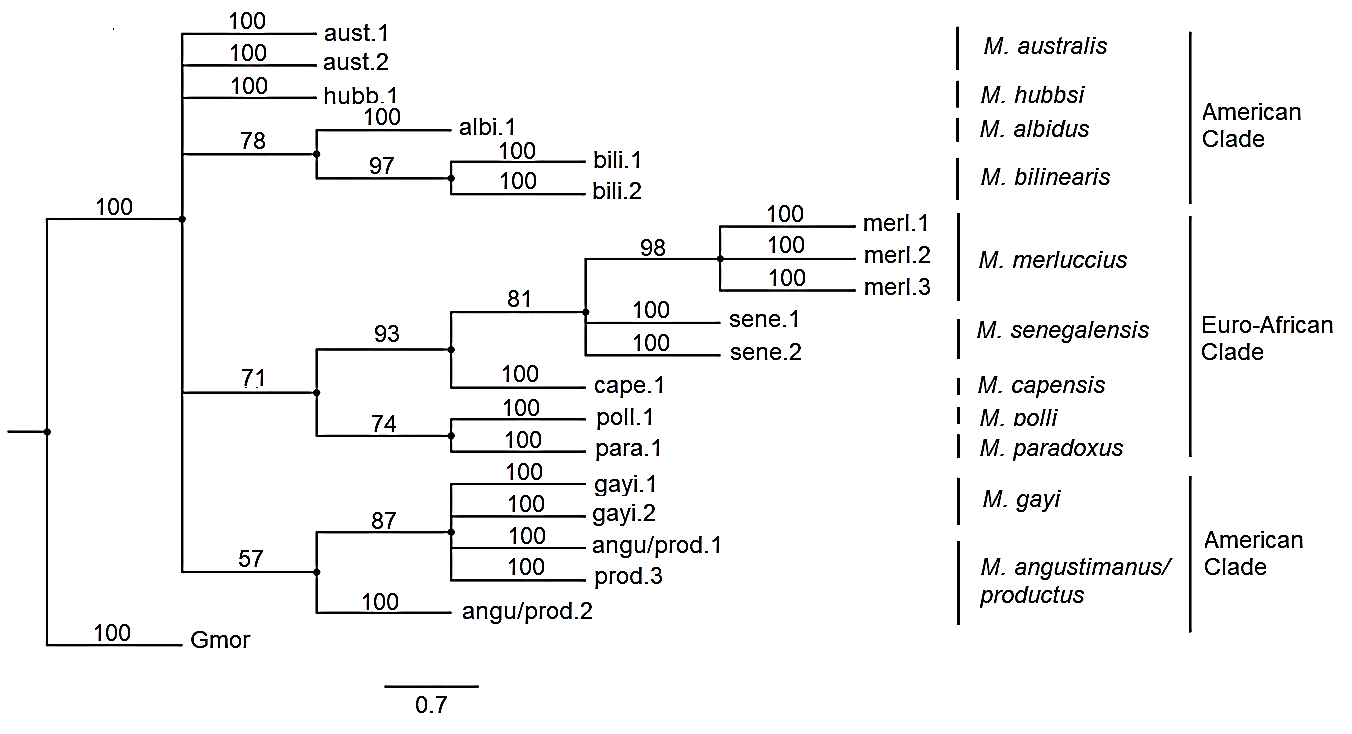

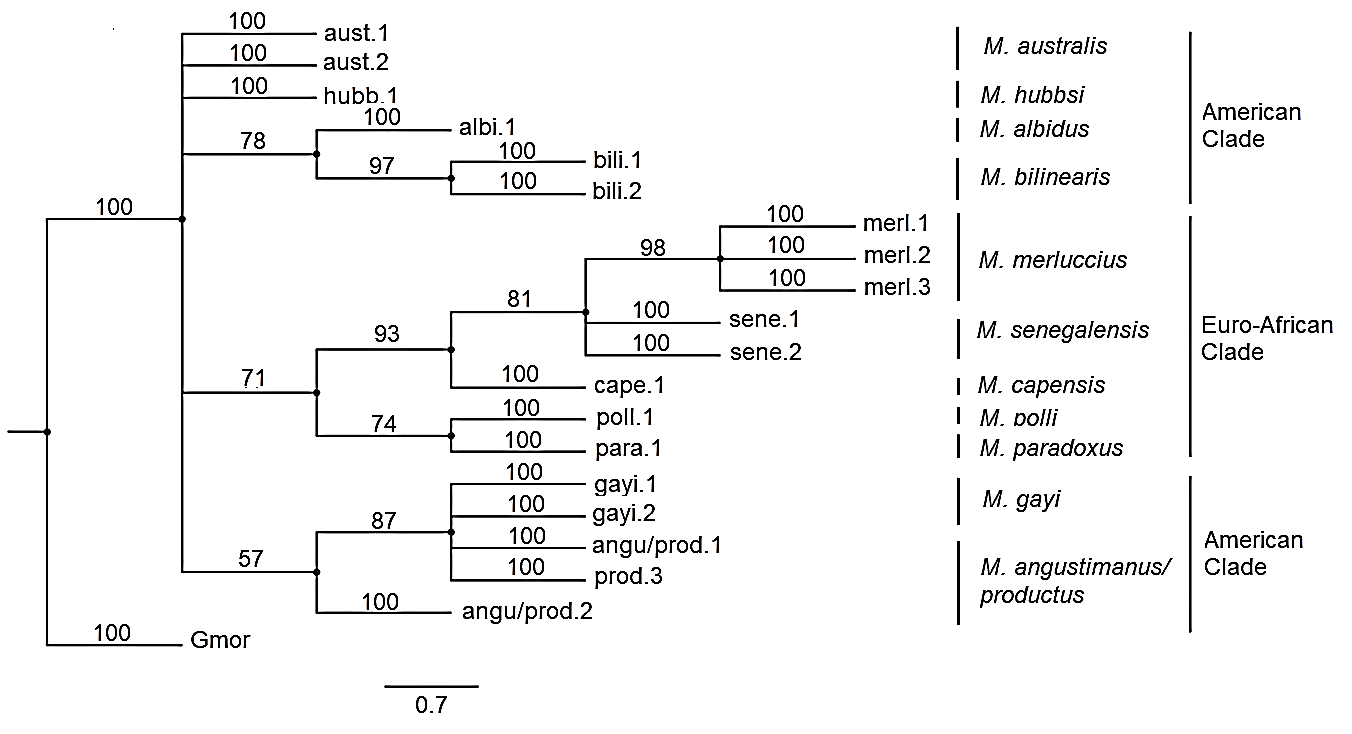
b**

**Supplementary Figure S2.** Phylogenetic reconstruction on ITS1 variants from genus *Merluccius* spp. using the substitution model HKY85+I+G. **a)** ML (-*lnL* = 1285.511) performed with PAUP v4.0 ^[84]^). Percentages of trees over 5000 bootstrap replicates are shown above branches; **b)** Bayesian reconstruction performed with MRBAYES v3.2.6 ^[87]^. Branches are annotated with bootstrap values, resp. percent posterior probabilities. Scale bar indicates the No. of nucleotide substitutions per 100 DNA residues. Sample codes are given in Table 1 and are followed by the number of the ITS1 variant per species (Supp. Table S3). The Atlantic cod (Gmor) was used as outgroup.

**
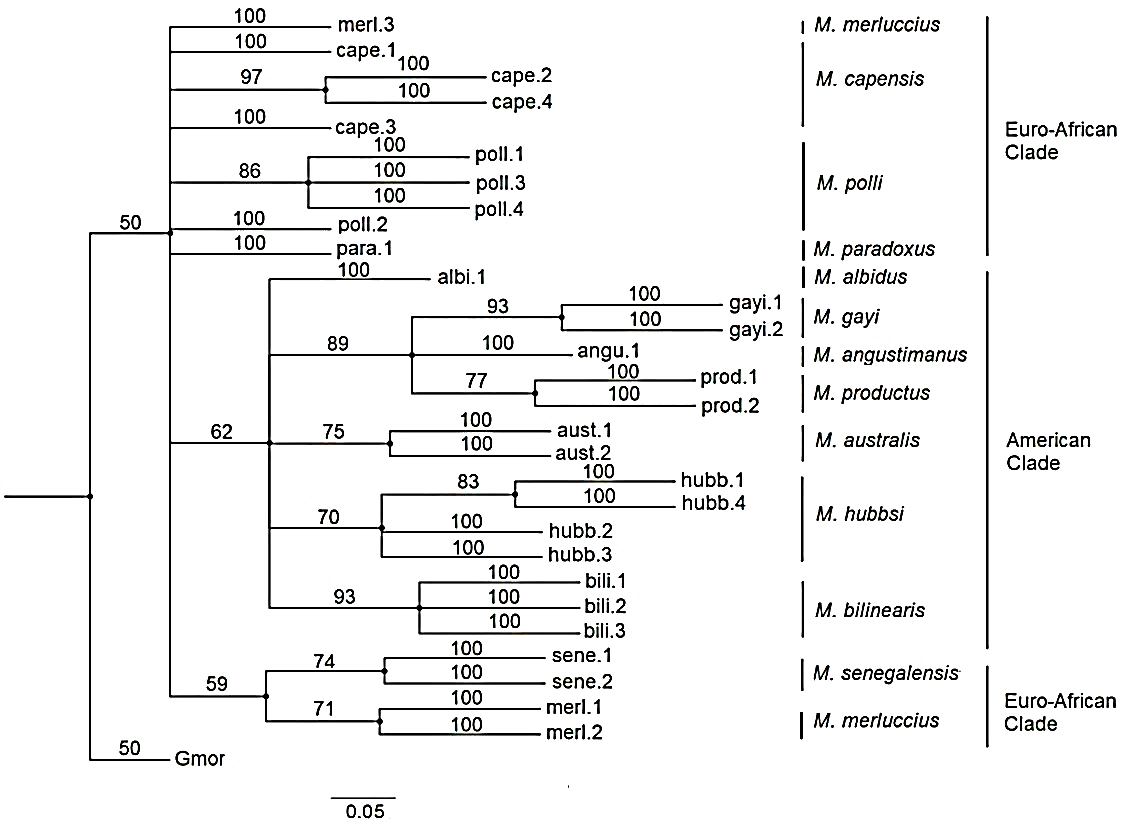
a**


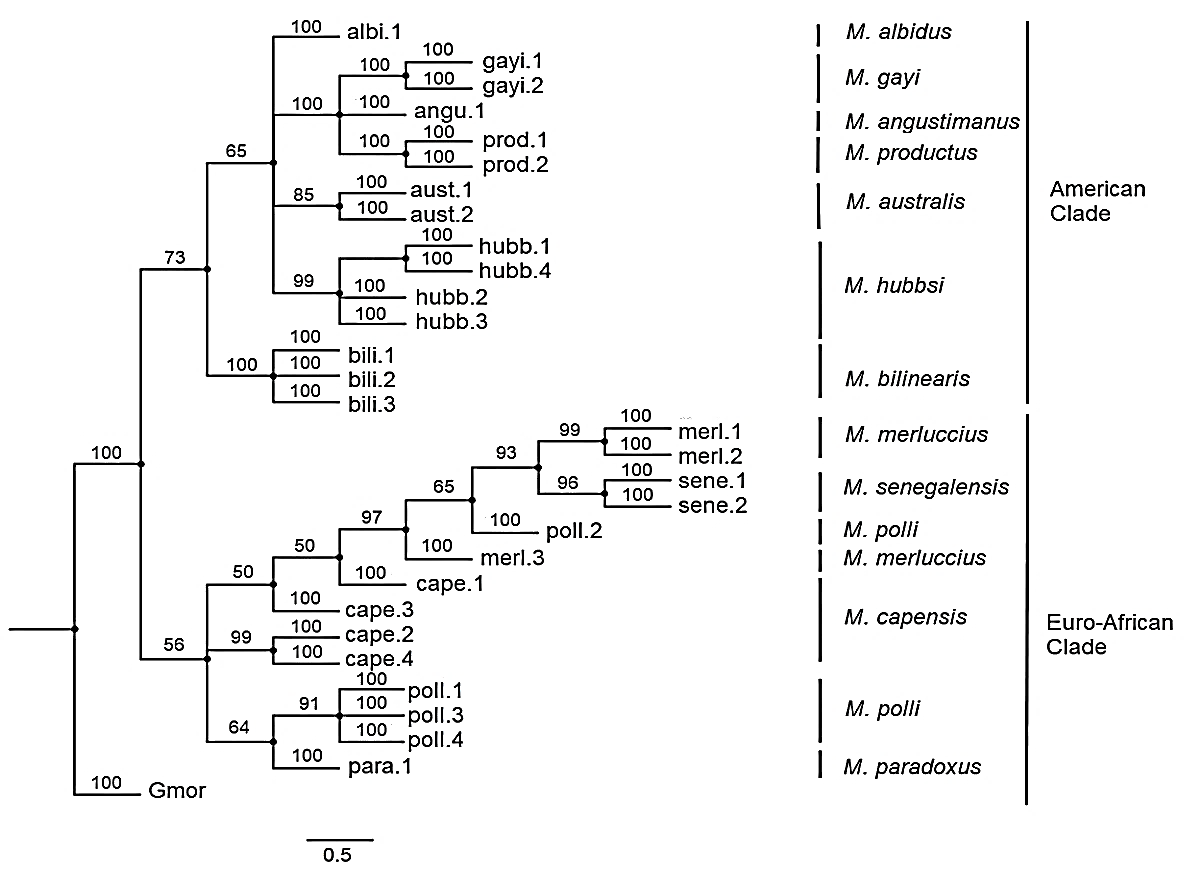
**b**

**Supplementary Figure S3**. Phylogenetic reconstruction on cyt b haplotypes from genus *Merluccius* spp. using the substitution model GTR+G. **a)** ML (-*lnL* = 1137.282) performed with PAUP v4.0 ^[84]^. Percentages of trees over 5000 bootstrap replicates are showed above branches; **b)** Bayesian reconstruction performed with MRBAYES v3.2.6 ^[87]^. Branches are annotated with bootstrap, resp. percent posterior probabilities. Scale bar indicates the No. of nucleotide substitutions per 100 DNA residues. Sample codes are given in Table 1 and are followed by the number of the cyt b haplotype per species (Supp. Table S4). The Atlantic cod (Gmor) was used as outgroup.

*
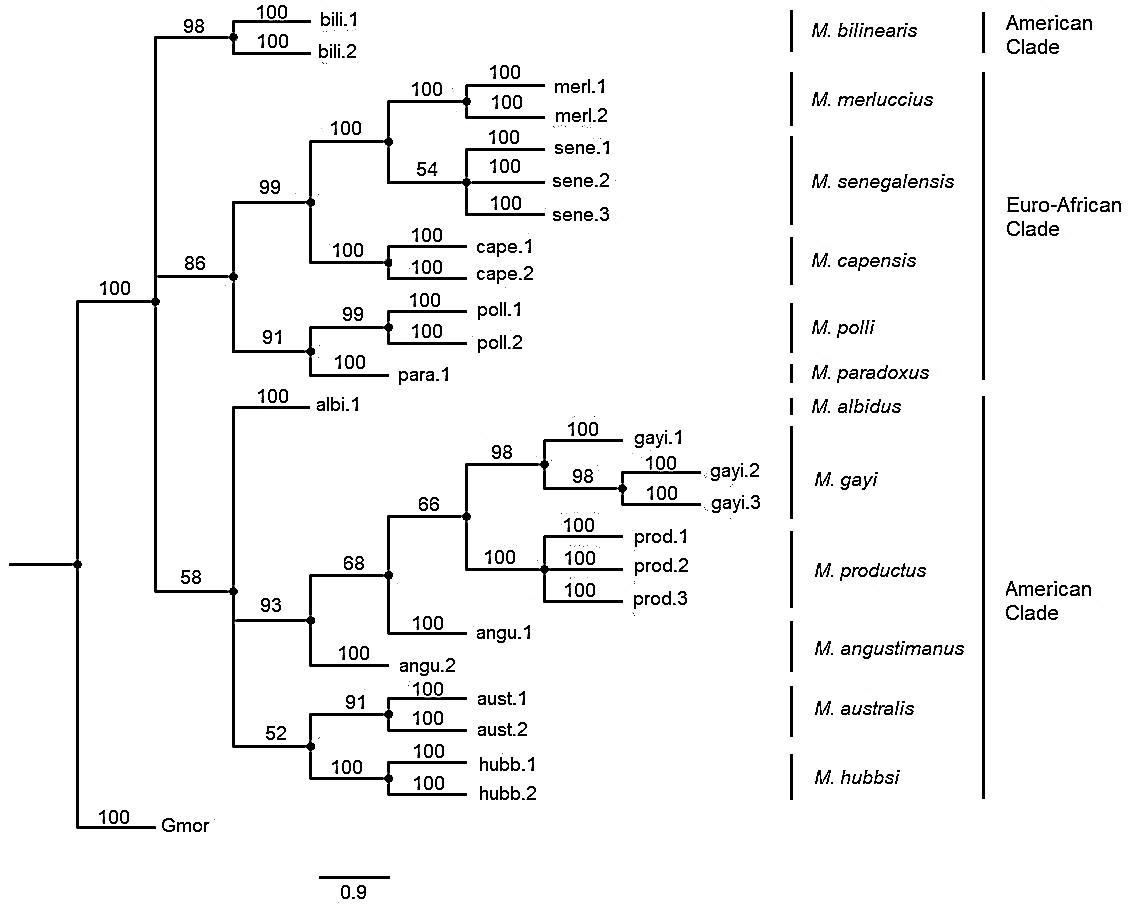
*

**Supplementary Figure S4.** Bayesian reconstruction performed with MRBAYES v3.2.6 ^[87]^ on 25 variants from 42 combined ITS1 *-* cyt b sequences of genus *Merluccius* spp. using the substitution model HKY85+I+G for ITS1 and GTR+G for cyt b. Branches are annotated with bootstrap, resp. percent posterior probabilities. Scale bar indicates the No. of nucleotide substitutions per 100 DNA residues. Sample codes are given in Table 1 and are followed by the number of the ITS1 *-* cyt b concatenated variants per species. The Atlantic cod (Gmor) was used as outgroup.
