# Supplementary material for: Phylogenetic prospecting for cryptic species of the genus Merluccius (Actinopterygii: Merlucciidae)
Source: Sci Rep. 2021 Mar 15;11:5929. doi: 10.1038/s41598-021-85008-9 (PMC7961003; doi:10.1038/s41598-021-85008-9)
Supplement: Supplementary file 2 — Supplementary Information 2. [file 41598_2021_85008_MOESM2_ESM.docx]

**Phylogenetic prospecting for cryptic species of the genus Merluccius (Actinopterygii: Merlucciidae)**

Montse Pérez^1^, María Fernández-Míguez^1, 2^, Jesús Matallanas^3^, Domingo Lloris^4^ and Pablo Presa^2,*^

^1^AquaCOV, Centro Oceanográfico de Vigo, Instituto Español de Oceanografía, 36390, Spain.

^2^CIM-REXENMAR, Universidad de Vigo, Facultad de Biología, 36310 Vigo, Spain.

^3^Unidad de Zoología, Departamento de Biología Animal, Biología Vegetal y Ecología, Universidad Autónoma de Barcelona, 08193, Spain.

^4^Institut de Ciències del Mar (CMIMA-CSIC), Barcelona, 08003, Spain

*[pressa@uvigo.es](mailto:pressa@uvigo.es)

**Supplementary Table S1.** Descriptors for the sequences used in the phylogenetic reconstruction of genus *Merluccius* with indication of their indexed codes.

| Data matrix | Sequence length including gaps (bp) | No. sequences | No.  variable sites | [No. of species]:  GenBank accession No. | Description |
| --- | --- | --- | --- | --- | --- |
| ITS1 | 692 | 85 | 254 | [12]: AY323942; AY323936; AY323941; AY323938; AY323937; AY323943; AY323945;  AY323944; AY323940;  AY323939; AY323946;  AY323947 | ITS1-rDNA complete sequences ^[33]^. |
| cyt b | 465 | 66 | 129 | [12]: AY821674; AY821673; AY821667; AY821675; AY821669; AY821664; AY821668; AY821665; AY821666; AY821672; AY821670; AY821671 | mtDNA-cyt b complete sequences ^[18]^. |
| ITS1 & cyt b | 1158 | 42 | 381 | (GenBank accession No. as above) | Specimens with sequence data from both, ITS1-rDNA and mtDNA-cyt b. |
| ITS1Nes | 71 | 85 | 13 | See ITS1Nes haplotypes in Table 3 | Specimens with sequence data from ITS1Nes – rDNA (46 clones of 14 specimens from 9 morphotypes plus 39 specimens from valid species). |

**Supplementary Table S2.** Intraspecific DNA sequence characteristics of ITS1 and cyt b from 11 valid hake species, *M. angustimanus* and *Gadus morhua* as computed with MEGA v7.0.20 ^[77]^. N, number of sequences; L, sequence length (bp); %GC, percentage of Guanine-Cytosine; *T_i_/T_v_*, Transition/Transversion ratio (*T_i_/T_v_* = ∞ when *T_v_* = 0 as indicated with “**–** “); Nv, number of nuclear sequence variants (ITS1) and haplotypes (cyt b); N.C., Nucleotide Composition (%).

|  | **ITS1** | | | | | |  | **Cyt b** | | | | | |
| --- | --- | --- | --- | --- | --- | --- | --- | --- | --- | --- | --- | --- | --- |
| **Species** | N | L | %GC | T_i_/T_v_ | Nv | N.C. (%) |  | N | L | %GC | T_i_/T_v_ | Nh | N. C. (%) |
| *M. merluccius* | 19 | 556 | 60.1 | - | 3 | 18.2T; 31.9C; 21.7A; 28.3G |  | 7 | 465 | 45.5 | 1.47 | 3 | 28.0T; 29.4C; 26.5A; 16.2G |
| *M. senegalensis* | 6 | 544 | 61.8 | 1.00 | 2 | 17.4T; 33.2C; 20.9A; 28.6G |  | 7 | 465 | 45.2 | - | 2 | 28.0T; 29.2C; 26.9A; 15.9G |
| *M. capensis* | 7 | 541 | 61.9 | - | 1 | 17.2T; 32.9C; 20.9A; 29.0G |  | 7 | 465 | 46.2 | 8.05 | 4 | 27.8T; 29.4C; 26.0A; 16.8G |
| *M. polli* | 5 | 587 | 63.1 | - | 1 | 18.8T; 32.7C; 18.2A; 30.4G |  | 5 | 465 | 44.6 | 4.28 | 4 | 28.2T; 28.9C; 27.2A; 15.7G |
| *M. paradoxus* | 2 | 557 | 60.9 | - | 1 | 18.7T; 32.4C; 20.4A; 28.6G |  | 4 | 465 | 45.4 | 0.00 | 1 | 28.2T; 29.7C; 26.5A; 15.7G |
| *M. albidus* | 3 | 536 | 61.3 | - | 1 | 18.3T; 33.4C; 20.3A; 28.0G |  | 3 | 464 | 48.5 | 0.00 | 1 | 26.1T; 31.5C; 25.4A; 17.0G |
| *M. gayi* | 8 | 531 | 61.0 | 5.10 | 2 | 18.4T; 33.5C; 20.5A; 27.5G |  | 7 | 464 | 47.4 | - | 2 | 25.6T; 31.3C; 26.9A; 16.2G |
| *M. productus* | 9 | 530 | 61.2 | 1.40 | 3 | 18.1T; 33.6C; 20.7A; 27.6G |  | 6 | 464 | 47.8 | - | 2 | 25.6T; 31.3C; 26.5A; 16.6G |
| *M. australis* | 9 | 539 | 61.7 | - | 2 | 17.6T; 34.3C; 20.6A; 27.5G |  | 4 | 464 | 48.0 | 1.00 | 2 | 26.0T; 31.7C; 26.0A; 16.3G |
| *M. hubbsi* | 6 | 529 | 63.4 | - | 1 | 18.2T; 34.5C; 18.3A; 28.9G |  | 4 | 464 | 48.5 | 1.26 | 4 | 25.5T; 31.8C; 26.0A; 16.8G |
| *M. bilinearis* | 7 | 537 | 61.4 | - | 2 | 17.8T; 34.4C; 20.8A; 27.1G |  | 7 | 464 | 48.1 | - | 3 | 25.4T; 31.3C; 26.4A; 16.9G |
| *M. angustimanus* | 3 | 528 | 60.9 | 0.00 | 2 | 18.1T; 33.1C; 21.0A; 27.7G |  | 4 | 464 | 47.2 | 0.00 | 1 | 25.6T; 31.3C; 27.2A; 15.9G |
| *Gadus morhua* | 1 | 532 | 63.0 | - | 1 | 17.1T; 32.9C; 19.9A; 30.1G |  | 1 | 464 | 40.9 | 0.00 | 1 | 33.0T; 25.2C; 26.1A; 15.7G |

**Supplementary Table S3.** ITS1 sequence variants and polymorphic sites as generated with DnaSP v5.10.1 ^[78]^ with indication of their absolute frequency per species. Species codes are given in Table 1.

|  |  | | | | | | | | | | | | |  | **Polymorphic sites** | | | | | |
| --- | --- | --- | --- | --- | --- | --- | --- | --- | --- | --- | --- | --- | --- | --- | --- | --- | --- | --- | --- | --- |
|  |  | | | | | | | | | | | | |  |  | 111111 | 1111111111 | 2222222222 | 2222222222 | 2222222222 |
|  | **Species** | | | | | | | | | | | | |  | 237778888 | 8888444444 | 5558999999 | 0000000011 | 1111333444 | 4444555555 |
| **Variants** | **merl** | **sene** | **cape** | **poll** | **para** | **albi** | **gayi** | **angu** | **prod** | **aust** | **hubb** | **bili** | **Gmor** |  | 7424890134 | 5679236789 | 4570024579 | 0123678914 | 5789789023 | 6789123456 |
| **HakeITS1.1** | 10 | - | - | - | - | - | - | - | - | - | - | - | - |  | GGTAGAGGTC | GAGTGTCCCG | AAAATGGAGT | AGCGCACCGT | GAGAGTCCCC | TTTTCTGACT |
| **HakeITS1.2** | 1 | - | - | - | - | - | - | - | - | - | - | - | - |  | ....A..... | .......... | .......... | .......... | .......... | .......... |
| **HakeITS1.3** | 1 | - | - | - | - | - | - | - | - | - | - | - | - |  | .......... | .......... | .......... | .......... | .......... | .....C.... |
| **HakeITS1.4** | - | 2 | - | - | - | - | - | - | - | - | - | - | - |  | .......... | .......... | .......... | ....T..... | .......... | ...C...... |
| **HakeITS1.5** | - | 4 | - | - | - | - | - | - | - | - | - | - | - |  | .......... | .......... | .......... | ....T..... | .......... | ...C...... |
| **HakeITS1.6** | - | - | 7 | - | - | - | - | - | - | - | - | - | - |  | .......... | .......... | .......... | ....T..... | .......... | ...C...... |
| **HakeITS1.7** | - | - | - | 5 | - | - | - | - | - | - | - | - | - |  | ...G...... | .......... | ...G...... | ....T..... | .......... | ...C.....C |
| **HakeITS1.8** | - | - | - | - | 5 | - | - | - | - | - | - | - | - |  | .......... | .......... | ..C....... | ....T..... | ........A. | ...C.....C |
| **HakeITS1.9** | - | - | - | - | - | 5 | - | - | - | - | - | - | - |  | T.C....... | .......... | .......... | ....T..... | .....A.... | ...C.C...C |
| **HakeITS1.10** | - | - | - | - | - | - | 7 | - | - | - | - | - | - |  | T.C....... | .......... | .......... | ....T..... | .....A.... | ...C.....C |
| **HakeITS1.11** | - | - | - | - | - | - | 1 | - | - | - | - | - | - |  | T.C....... | .......... | .......... | ....T..... | .....A.... | ...C.....C |
| **HakeITS1.12** | - | - | - | - | - | - | - | 4 | 6 | - | - | - | - |  | T.C....... | .......... | .......... | ....T..... | .....A.... | ...C.....C |
| **HakeITS1.13** | - | - | - | - | - | - | - | 2 | 2 | - | - | - | - |  | T.C....... | .......... | .......... | ....T..... | .....A.... | ...C.....C |
| **HakeITS1.14** | - | - | - | - | - | - | - | - | 1 | - | - | - | - |  | T.C....... | .......... | .......... | ....T..... | .....A.... | ...C.....C |
| **HakeITS1.15** | - | - | - | - | - | - | - | - | - | 8 | - | - | - |  | T.C....... | .......... | .......... | ....T..... | .....A.... | ...C.C.... |
| **HakeITS1.16** | - | - | - | - | - | - | - | - | - | 1 | - | - | - |  | TAC....... | .......... | .......... | ....T..... | .....A.... | ...C.C.... |
| **HakeITS1.17** | - | - | - | - | - | - | - | - | - | - | 6 | - | - |  | T.C....... | .......... | .......... | ....T..... | .......... | ...C.C.... |
| **HakeITS1.18** | - | - | - | - | - | - | - | - | - | - | - | 6 | - |  | TTC....... | .......... | .......... | ....A..... | .....A.... | ...C.C...C |
| **HakeITS1.19** | - | - | - | - | - | - | - | - | - | - | - | 1 | - |  | TTC....... | .......... | .......... | ....A..... | .....A.... | ...C.C...C |
| **GmorITS1.20** | - | - | - | - | - | - | - | - | - | - | - | - | 1 |  | TTACACACGT | TGCCAAGTGA | GGCCCCAGAA | GCTCTCAACC | CGAGCGCTGT | GGAGGGAGTG |

| **Variants** | **Polymorphic sites** | | | | | | | | | | | | |
| --- | --- | --- | --- | --- | --- | --- | --- | --- | --- | --- | --- | --- | --- |
|  | 2222222222 | 2222222222 | 3333333333 | 3333333333 | 3333333333 | 3333333333 | 3333333444 | 4444444444 | 4444444444 | 4444444444 | 4444445555 | 5555555555 | 5555555555 |
|  | 5566666778 | 8889999999 | 0000000011 | 1111223333 | 3344445555 | 5566677778 | 8899999000 | 0111122222 | 3333346667 | 7777888888 | 9999990000 | 0111111222 | 2223333333 |
|  | 7812357492 | 4572456789 | 1234567892 | 3678890146 | 8912790127 | 8967801242 | 8901236346 | 8078934579 | 2356953561 | 2789056789 | 0347890246 | 9013456702 | 5679012346 |
| **HakeITS1.1** | CTTTGCCTCG | ACCTTCCGAG | TCTGACGGGG | TGGCTCCGAA | GCCTCTCAGT | GATGTTCAAG | TCCTATCTGT | ACTAAGCACC | TGCCGGTGCC | TGGTAGCCAC | ATTGCGTTCT | GCAGCGGGGG | GTTAATGACG |
| **HakeITS1.2** | .......... | .......... | .......... | .......... | .......... | .......... | .......... | .......... | .......... | .......... | .......... | .......... | .......... |
| **HakeITS1.3** | .......... | ...C...... | .......... | .......... | .......... | .......... | .......... | .......... | .......... | .......... | .......... | .......... | .......... |
| **HakeITS1.4** | .......... | .......... | ..C....... | .......... | .......... | .......... | .......... | ...T...... | .......... | .......... | .......... | .......... | .......... |
| **HakeITS1.5** | .......... | .......... | ..C....... | .......... | .......... | .......... | .......... | .......... | .......... | .......... | .......... | .......... | .......... |
| **HakeITS1.6** | .......... | .......... | ..C....... | .......... | .......... | .......... | .......... | .......... | .......... | .......... | .C........ | .......... | .......... |
| **HakeITS1.7** | .......... | .......... | ..A..G..A. | ...G..AAG. | .......G.. | .G........ | .G........ | .......G.. | C.T.C..... | .....T.... | .C..G..... | .....C.... | .......... |
| **HakeITS1.8** | .C....T... | .......... | .AA..G..A. | ....C...G. | .......... | .G........ | .G........ | ..C....G.. | C.T.T..... | .....T.... | .C..G..... | .....C.... | .......... |
| **HakeITS1.9** | .......... | .......T.. | .A...G..A. | ........G. | .........A | .G........ | .G........ | ..C..T.... | C.T.T..... | ......T... | .C..G..... | .....C.... | .......... |
| **HakeITS1.10** | .C........ | .......... | .AC..G..A. | ........G. | .........A | .G........ | .G........ | ..C..T.... | C...T..... | ......T... | .C..G..... | .....C...T | .......... |
| **HakeITS1.11** | .C........ | .......... | .AC..G..A. | ........G. | ..T......A | .G........ | .G........ | ..C..T.... | C...T....G | ......T... | .C..G..... | .....C...T | .......... |
| **HakeITS1.12** | .C........ | .......... | .AC..G..A. | ........G. | .........A | .G........ | .G........ | ..C..T.... | C...T..... | ......T... | .C..G..... | .....C...T | .......... |
| **HakeITS1.13** | .C........ | .......... | .AC..G..A. | ........G. | .........A | .G........ | .G........ | ..C..T.... | C...T..... | ......T... | .C..G..... | .....C.... | .......... |
| **HakeITS1.14** | .C........ | .......... | .AC..G..A. | ........G. | .........A | .G........ | .G........ | G.C..T.... | C...T..... | ......T... | .C..G..... | .....C...T | .......... |
| **HakeITS1.15** | .C........ | .......... | .AC..G..A. | ........G. | .........A | .G........ | .G........ | ..C..T.... | C...T..... | ......T... | .C..G..... | .....C.... | .......... |
| **HakeITS1.16** | .C........ | .......... | .AC..G..A. | ........G. | .........A | .G........ | .G........ | ..C..T.... | C...T..... | ......T... | .C..G..... | .....C.... | .......... |
| **HakeITS1.17** | .C........ | G......... | .AC..G..A. | C.......G. | ........CG | .G........ | .G........ | ..C..T.... | C...T..... | ......T... | .C..G..... | .....C.... | .......... |
| **HakeITS1.18** | .......... | .......C.. | .A...G..A. | ....C...G. | .........A | .G........ | .G........ | C.C..T.... | C.T.T..... | ......T... | .C..G..... | .....C.... | .......... |
| **HakeITS1.19** | .......... | .......C.. | .A...G..A. | ....C...G. | .........A | .G........ | .G........ | C.C..T.... | C.T.T..A.. | ......T... | .C..G..... | .....C.... | .......... |
| **GmorITS1.20** | AACCCA.GGA | GTACCTACCA | CCCACCCCGC | CACTTGACGC | TTTCGCTGAG | C.GCAGGCTT | GGAGGCTAAC | TGCGTTGGAA | CCGGTCAAAA | ACAGCCGGTG | TGATGACGAC | TGTCGCTCC. | TACCTAACAA |

…cont. **Supplementary Table S3**…

| **Variants** | **Polymorphic sites** | | | | | | |
| --- | --- | --- | --- | --- | --- | --- | --- |
|  | 5555555555 | 5555555555 | 5555555555 | 5555555666 | 6666666666 | 6666666666 | 6666 |
|  | 3444444555 | 5555566666 | 6677777777 | 8888899033 | 3333344444 | 4444555555 | 6679 |
|  | 8012478134 | 5678903567 | 8912345789 | 1234557312 | 4568901245 | 6789124569 | 2322 |
| **HakeITS1.1** | GGTGTACGTT | CGGTCATGTC | CACGCGGGCC | GTAAGTTATC | AATATCGAGC | TTTGCCAAAC | AACA |
| **HakeITS1.2** | .......... | .......... | .......... | .......... | .......... | .......... | .... |
| **HakeITS1.3** | .......... | .......... | .......... | .......... | .......... | .......... | .... |
| **HakeITS1.4** | .......... | .......... | .......... | .......... | ...G...... | .......... | .... |
| **HakeITS1.5** | .......... | .......... | .......... | .......... | ...G...... | .......... | .... |
| **HakeITS1.6** | .......... | .......... | .......... | .......... | ...G...... | .......... | .... |
| **HakeITS1.7** | .......... | ...C...... | .......... | .......... | ...G....C. | .......... | .... |
| **HakeITS1.8** | .......... | .......... | .......... | .......... | ........C. | .......... | .... |
| **HakeITS1.9** | .......... | .......... | .......... | ..C.....AT | G..G....C | .......... | .... |
| **HakeITS1.10** | .......... | .......... | .......... | ..CC....AT | G..G....C. | .......... | .... |
| **HakeITS1.11** | .......... | .......... | .......... | ..C.....AT | G..G....C. | .......... | .... |
| **HakeITS1.12** | .......... | .......... | .......... | ..C.....AT | G..G....C. | .......... | .... |
| **HakeITS1.13** | .......... | .......... | .......... | ..C.....AT | G..G....C. | .......... | .... |
| **HakeITS1.14** | .......... | .......... | .......... | ..C.....AT | G..G....C. | .......... | .... |
| **HakeITS1.15** | .......... | .......... | .......... | ..C.....AT | G..G....C. | .......... | .... |
| **HakeITS1.16** | .......... | .......... | .......... | ..C.....AT | G..G....C. | .......... | .... |
| **HakeITS1.17** | .AC....... | .......... | .G........ | ..CC.....T | G..G....C. | .......... | .... |
| **HakeITS1.18** | .......... | .......... | .......... | ..C......T | G..G...... | .......... | .... |
| **HakeITS1.19** | .......... | .......... | .......... | ..C......T | G..G...... | .......... | .... |
| **GmorITS1.20** | TTCCCCTTAC | GCAGGTGCGA | GGGTTCATGA | CGCGTGGG.T | GCCCGTCGTA | CAAARGCTTA | TCGG |

…cont. **Supplementary Table S3**.

**Supplementary Table S4.** Cyt b haplotypes and polymorphic sites as generated with DnaSP v5.10.1 ^[78]^ with indication of their absolute frequency per species. Species codes are given in Table 1.

|  |  | | | | | | | | | | | | | **Polymorphic sites** | | | | | |
| --- | --- | --- | --- | --- | --- | --- | --- | --- | --- | --- | --- | --- | --- | --- | --- | --- | --- | --- | --- |
|  |  | | | | | | | | | | | | |  | 1 | 1111111111 | 1111111111 | 1111111111 | 1111222222 |
|  | **Species** | | | | | | | | | | | | | 3455666677 | 7888888990 | 0011112222 | 2334455566 | 6667778888 | 9999000111 |
| **Haplotypes** | merl | sene | cape | poll | para | albi | gayi | angu | prod | aust | hubb | bili | Gmor | 4928145703 | 6234589470 | 3923561234 | 6695647812 | 3460231248 | 0369269279 |
| **HakeCytb.1** | 5 | - | - | - | - | - | - | - | - | - | - | - | - | CCATTCCTAG | TTGATATACT | TCTCCAACAT | CCGCCCCTGC | CGCACTGCAC | AGATTGAGGT |
| **HakeCytb.2** | 1 | - | - | - | - | - | - | - | - | - | - | - | - | .......... | .......... | .......... | .......... | .......... | ...C...... |
| **HakeCytb.3** | 1 | - | - | - | - | - | - | - | - | - | - | - | - | .......... | .......... | ....A....G | .T........ | ......A..T | .......... |
| **HakeCytb.4** | - | 6 | - | - | - | - | - | - | - | - | - | - | - | .......... | .......... | ....A..... | .......... | ........G. | .A......A. |
| **HakeCytb.5** | - | 1 | - | - | - | - | - | - | - | - | - | - | - | .......... | .......... | ....A..... | .......... | ........G. | .A......A. |
| **HakeCytb.6** | - | - | 1 | - | - | - | - | - | - | - | - | - | - | .......... | .......... | ..C.A...G. | .T..T..... | ......A..T | .A.CC..... |
| **HakeCytb.7** | - | - | 4 | - | - | - | - | - | - | - | - | - | - | .......C.. | .......... | ..C.A....G | .T..T..... | ......CT.. | G..CC...A. |
| **HakeCytb.8** | - | - | 1 | - | - | - | - | - | - | - | - | - | - | .......... | .......... | ..C.A...G. | .T..T..... | ......A..T | .A.CC..... |
| **HakeCytb.9** | - | - | 1 | - | - | - | - | - | - | - | - | - | - | .......C.. | .......... | ..C.A....G | .T..T..... | ......CT.. | G..CC...A. |
| **HakeCytb.10** | - | - | - | 2 | - | - | - | - | - | - | - | - | - | ....CT.... | .......... | ..C.A....G | .T.TT..... | ......A..T | .A.C....A. |
| **HakeCytb.11** | - | - | - | 1 | - | - | - | - | - | - | - | - | - | .......... | .......... | ....A..... | .......... | ......A... | .A......A. |
| **HakeCytb.12** | - | - | - | 1 | - | - | - | - | - | - | - | - | - | ....CT.... | .......... | ..C.A....G | .T.TT..... | ......A..T | .A.C....A. |
| **HakeCytb.13** | - | - | - | 1 | - | - | - | - | - | - | - | - | - | ....CT.... | .......... | ..C.A....G | .T.TT..... | ......A..T | .A.C....A. |
| **HakeCytb.14** | - | - | - | - | 4 | - | - | - | - | - | - | - | - | ....C..... | .......... | ..C.A...G. | .T.TT..... | ......A..T | .A.CC..... |
| **HakeCytb.15** | - | - | - | - | - | 3 | - | - | - | - | - | - | - | ...CC..C.. | CC.......C | ..C.AG...G | .TA..TTC.. | ......A... | G..CC...A. |
| **HakeCytb.16** | - | - | - | - | - | - | 6 | - | - | - | - | - | - | ...CC..C.. | C........C | ..C.AC...G | .T....TC.. | ......A... | .A.CC...A. |
| **HakeCytb.17** | - | - | - | - | - | - | 1 | - | - | - | - | - | - | ...CC..C.. | C........C | ..C.AC...G | .T....TC.. | ......A... | ...CC...A. |
| **HakeCytb.18** | - | - | - | - | - | - | - | 4 | - | - | - | - | - | ...CC..C.. | C........C | ..C.AC...G | .TA...TC.. | ......A... | .A.CC...A. |
| **HakeCytb.19** | - | - | - | - | - | - | - | - | 5 | - | - | - | - | ...CC..C.. | C........C | ..C.AC...G | .T....TC.. | ......A... | .A.CC...A. |
| **HakeCytb.20** | - | - | - | - | - | - | - | - | 1 | - | - | - | - | ...CC..C.. | C........C | ..C.AC...G | .T....TC.. | .A....A... | .A.CC...A. |
| **HakeCytb.21** | - | - | - | - | - | - | - | - | - | 3 | - | - | - | ...CC..CG. | C........C | ..C.A.G..G | .T....TC.. | ......A... | .A.CC...A. |
| **HakeCytb.22** | - | - | - | - | - | - | - | - | - | 1 | - | - | - | ...CC..C.. | C........C | ..C.A.G..G | .T....TC.. | ......A... | .A.CC...A. |
| **HakeCytb.23** | - | - | - | - | - | - | - | - | - | - | 1 | - | - | ...CC..... | C....GG..C | .TC.AG...G | .T....TC.. | ......A... | .A.CC...A. |
| **HakeCytb.24** | - | - | - | - | - | - | - | - | - | - | 1 | - | - | ...CC..... | C....GG..C | .TC.A.G..G | .T....TC.. | ......A... | .A.CC...A. |
| **HakeCytb.25** | - | - | - | - | - | - | - | - | - | - | 1 | - | - | ...CC..... | C....GG..C | .TC.A....G | .T....TC.. | ......A... | .A.CC...A. |
| **HakeCytb.26** | - | - | - | - | - | - | - | - | - | - | 1 | - | - | ...CC..... | C....GG..C | .TC.A....G | .T....TC.. | ......A... | .A.CC...A. |
| **HakeCytb.27** | - | - | - | - | - | - | - | - | - | - | - | 5 | - | ...CC..C.. | C...C....G | ..C.AG...G | .T.TT.TC.. | G....CA..T | .A.CC...A. |
| **HakeCytb.28** | - | - | - | - | - | - | - | - | - | - | - | 1 | - | ...CC..C.. | C...C....G | ..C.AG..GG | .T.TT.TC.. | G....CA..T | .A.CC...A. |
| **HakeCytb.29** | - | - | - | - | - | - | - | - | - | - | - | 1 | - | ...CC..C.. | C...C....G | ..C.AG...G | .T.TT.TC.. | G....CA..T | .AGCC...A. |
| **GmorCytb.30** | - | - | - | - | - | - | - | - | - | - | - | - | 1 | TTGC.AAC.A | ..AGC..TTC | C.CTA.GT.G | TTCT.T..AT | TATCTCA... | ...C.TGA.C |

…cont. **Supplementary Table S4**

| **Haplotypes** | **Polymorphic sites** | | | | | | |  |
| --- | --- | --- | --- | --- | --- | --- | --- | --- |
|  | 2222222222 | 2222222222 | 2233333333 | 3333333333 | 3333333333 | 3333444444 | 444444444 | |
|  | 2233345555 | 6667788889 | 9900111122 | 2334444555 | 5666677788 | 8999000011 | 112333356 | |
|  | 3925870369 | 2584703692 | 5817036293 | 5140369285 | 9145703925 | 8147013602 | 687036913 | |
| **HakeCytb.1** | TACCACCCAT | CAACCCACCC | CTTCCCCCCC | ATACATCCCC | TACAAGCCTA | TAACTTAAAG | GCCCCCTAC | |
| **HakeCytb.2** | .......... | .......... | .......... | .......... | .......... | .......... | ......... | |
| **HakeCytb.3** | .......... | .......... | .......G.. | ........T. | .......... | .......... | ......... | |
| **HakeCytb.4** | .......... | .......... | .......... | .......... | .......... | .......... | ......... | |
| **HakeCytb.5** | .......... | .......... | .......... | .......... | .......... | .......... | .......G. | |
| **HakeCytb.6** | ........G. | .......... | .......... | ........T. | .......... | .....C.... | ....T.... | |
| **HakeCytb.7** | .......... | .......... | .......... | G.......T. | ..T.....CG | ..G....... | ..T...CG. | |
| **HakeCytb.8** | ........G. | .......... | .......... | ........T. | ..T.....C. | .....C.... | ......... | |
| **HakeCytb.9** | .......... | .......... | .......... | G.......T. | ..T.....CG | ..G....... | ......CG. | |
| **HakeCytb.10** | C......... | ...T...... | T......A.. | ....CC..T. | ..T.....CC | ....CC...A | .T.T..C.. | |
| **HakeCytb.11** | .......... | .......... | .......G.. | .....C..T. | ..T....... | .....C.... | .T.T..... | |
| **HakeCytb.12** | C......... | ...T..G... | T......G.. | ....CC..T. | ..T.....CC | ....CC...A | .T.T..C.. | |
| **HakeCytb.13** | C......... | ...T...... | T......G.. | ....CC..T. | ..T.....CC | ....CC...A | .T.T..C.. | |
| **HakeCytb.14** | ........G. | .C.T...... | T......... | C....C..T. | ..T..A..CC | ....CC...A | ...TT.C.. | |
| **HakeCytb.15** | C..T...... | T..T.T.... | TCC.T..... | CC..GC.... | CGT.G...C. | C.T.CC..GC | ..T.TTCG. | |
| **HakeCytb.16** | C.AT..T... | ..GT.T.... | .CC.T..... | .C.T.C.... | CGT.GA..C. | ..C.CC...A | ..T..ACG. | |
| **HakeCytb.17** | C.AT..T... | ..GT.T.... | .CC.T..... | .C.T.C.... | CGT.GA..C. | ..C.CC...A | ..T..ACG. | |
| **HakeCytb.18** | C.AT..T... | ...T.T.... | .CC.T..... | .C.TGC.... | CGT.GA..C. | C.C.CC...A | ..T.TACG. | |
| **HakeCytb.19** | C.AT..T... | .G.T.T.... | .CC.T..... | .C.TGC.... | CGT.GA..C. | C.C.CCG..A | ..T.TACG. | |
| **HakeCytb.20** | C.AT..T... | .G.T.T.... | .CC.T..... | .C.TGC.... | CGT.GA..C. | C.C.CCG..A | ..T.TACG. | |
| **HakeCytb.21** | C..TT..... | TG.T.T.... | .CC.T..... | .C..GC.... | CCT.GA..C. | ..T.CC...C | ..T.T.C.. | |
| **HakeCytb.22** | C..T...... | TG.T.T.... | .CC.T..... | .C..GC.... | CCT.GA..C. | ..T.CC...C | ..T.T.C.. | |
| **HakeCytb.23** | CC.T...... | ...T...... | .CC.T..... | .C..GC.... | CGT.G..TC. | C.T.CCG.GC | ....T.C.. | |
| **HakeCytb.24** | C..TT..... | ...T...... | .CC.T..... | .C..GC.... | CCT.GA.TC. | C.T.CC...C | ....T.C.. | |
| **HakeCytb.25** | C..T...... | ...T...... | .CC.T..... | .C..GC.... | C.T.G..TC. | C.T.CC.... | ....T.C.. | |
| **HakeCytb.26** | CC.T...... | ...T...... | .CC.T..... | .C..GC.... | C.T.G..TC. | C.T.CCG.GC | ....T.C.. | |
| **HakeCytb.27** | C......... | T..T...... | .CC.T....A | .C...C.... | C.T.GA...G | C.CTCC.T.. | ......CG. | |
| **HakeCytb.28** | C......... | T..T...... | .CC.T....A | .C...C.... | C.T.GA...G | C.CTCC.T.. | ......CG. | |
| **HakeCytb.29** | C......... | T..T...... | .CC.T....A | .C...C.... | C.T.GA...G | C.CTCC.T.. | ......CG. | |
| **GmorCytb.30** | C..ACTTT.C | .C.TGT.TTT | TC.T.TTTTA | G.CTC.TT.T | CTTG..A.CG | .CT......A | TTTT..C.A | |

**Supplementary Table S5.** Intraspecific nucleotide diversity of ITS1 sequences (*P_i_* *± S.D*., on the diagonal), average number of nucleotide substitutions per site between species (*D*_xy_ ± *S.D.*, below the diagonal) as generated with DnaSP v5.10.1 ^[78]^ and Net Value of Evolutionary Divergence between species (*d*, above the diagonal) as generated with MEGA v7.0.20 ^[77]^. Dotted lines separate pairwise comparisons within and between species from Euro-Africa (Old World hakes) and America (New World hakes). Species codes are given in Table 1.

|  | merl | sene | cape | poll | para | albi | gayi | angu | prod | aust | hubb | bili | Gmor |
| --- | --- | --- | --- | --- | --- | --- | --- | --- | --- | --- | --- | --- | --- |
| merl | 0.001±0.001 | 0.013 | 0.015 | 0.078 | 0.075 | 0.090 | 0.094 | 0.089 | 0.090 | 0.087 | 0.103 | 0.094 | 2.638 |
| sene | 0.017±0.017 | 0.002±0.001 | 0.004 | 0.069 | 0.072 | 0.085 | 0.082 | 0.078 | 0.078 | 0.076 | 0.091 | 0.092 | 2.606 |
| cape | 0.022±0.009 | 0.005±0.002 | 0.000±0.000 | 0.065 | 0.068 | 0.081 | 0.078 | 0.078 | 0.074 | 0.072 | 0.087 | 0.087 | 2.595 |
| poll | 0.083±0.088 | 0.072±0.035 | 0.075±0.042 | 0.000±0.000 | 0.046 | 0.074 | 0.081 | 0.076 | 0.077 | 0.081 | 0.096 | 0.088 | 2.323 |
| para | 0.076±0.079 | 0.071±0.039 | 0.073±0.055 | 0.052±0.037 | 0.000±0.000 | 0.062 | 0.062 | 0.058 | 0.058 | 0.062 | 0.077 | 0.069 | 2.353 |
| albi | 0.081±0.043 | 0.081±0.043 | 0.076±0.049 | 0.084±0.057 | 0.065±0.048 | 0.000±0.000 | 0.020 | 0.016 | 0.017 | 0.014 | 0.043 | 0.020 | 2.190 |
| gayi | 0.075±0.027 | 0.072±0.031 | 0.071±0.034 | 0.075±0.000 | 0.064±0.066 | 0.029±0.013 | 0.003±0.001 | 0.004 | 0.004 | 0.012 | 0.036 | 0.038 | 2.266 |
| angu | 0.080±0.042 | 0.075±0.035 | 0.072±0.044 | 0.075±0.046 | 0.066±0.069 | 0.029±0.029 | 0.009±0.004 | 0.004±0.001 | 0.001 | 0.008 | 0.037 | 0.034 | 2.236 |
| prod | 0.074±0.024 | 0.071±0.074 | 0.068±0.028 | 0.075±0.034 | 0.064±0.067 | 0.025±0.012 | 0.004±0.002 | 0.006±0.003 | 0.000±0.000 | 0.008 | 0.037 | 0.034 | 2.241 |
| aust | 0.071±0.071 | 0.066±0.028 | 0.064±0.029 | 0.077±0.081 | 0.058±0.038 | 0.024±0.013 | 0.011±0.004 | 0.015±0.008 | 0.018±0.009 | 0.001±0.000 | 0.029 | 0.032 | 2.202 |
| hubb | 0.090±0.096 | 0.084±0.041 | 0.082±0.045 | 0.094±0.055 | 0.078±0.058 | 0.049±0.032 | 0.034±0.014 | 0.044±0.045 | 0.034±0.014 | 0.028±0.013 | 0.000±0.000 | 0.056 | 2.081 |
| bili | 0.093±0.034 | 0.087±0.032 | 0.084±0.037 | 0.086±0.039 | 0.074±0.039 | 0.041±0.019 | 0.044±0.014 | 0.039±0.017 | 0.039±0.013 | 0.042±0.016 | 0.059±0.027 | 0.001±0.000 | 2.138 |
| Gmor | 0.703±1.805 | 0.698±1.361 | 0.694±1.801 | 0.691±1.902 | 0.701±1.446 | 0.679±1.445 | 0.683±1.497 | 0.682±1.094 | 0.683±1.219 | 0.683±1.529 | 0.673±1.558 | 0.682±1.448 | 0.000±0.000 |

**Supplementary Table S6.** Intraspecific nucleotide diversity of cyt b sequences (*P_i_* ± *S.D*., on the diagonal), average number of nucleotide substitutions per site between species (*D*_xy_ ± *S.D*., below the diagonal) as generated with DnaSP v5.10.1 ^[78]^ and Net Value of Evolutionary Divergence between species (*d*, above the diagonal) as generated with MEGA v7.0.20 ^[77]^. Dotted lines separate pairwise comparisons within and between hakes from Euro-Africa (Old World) and America (New World). The species *M. merluccius* is included as a relative measure of divergence. Species codes are given in Table 1.

|  | merl | sene | cape | poll | para | albi | gayi | angu | prod | aust | hubb | bili | Gmor |
| --- | --- | --- | --- | --- | --- | --- | --- | --- | --- | --- | --- | --- | --- |
| merl | 0.004±0.003 | 0.006 | 0.023 | 0.034 | 0.047 | 0.085 | 0.075 | 0.082 | 0.084 | 0.074 | 0.067 | 0.078 | 0.173 |
| sene | 0.011±0.004 | 0.001±0.001 | 0.025 | 0.033 | 0.049 | 0.087 | 0.073 | 0.079 | 0.081 | 0.072 | 0.065 | 0.075 | 0.178 |
| cape | 0.041±0.015 | 0.041±0.016 | 0.018±0.007 | 0.027 | 0.033 | 0.065 | 0.059 | 0.065 | 0.066 | 0.060 | 0.058 | 0.059 | 0.160 |
| poll | 0.054±0.023 | 0.051±0.024 | 0.052±0.018 | 0.018±0.009 | 0.020 | 0.070 | 0.060 | 0.064 | 0.066 | 0.058 | 0.051 | 0.057 | 0.151 |
| para | 0.062±0.049 | 0.063±0.054 | 0.051±0.037 | 0.035±0.018 | 0.000±0.000 | 0.076 | 0.068 | 0.070 | 0.070 | 0.063 | 0.061 | 0.064 | 0.171 |
| albi | 0.105±0.058 | 0.105±0.063 | 0.088±0.042 | 0.094±0.046 | 0.093±0.081 | 0.000±0.000 | 0.035 | 0.027 | 0.032 | 0.028 | 0.031 | 0.052 | 0.178 |
| gayi | 0.094±0.039 | 0.091±0.041 | 0.082±0.032 | 0.082±0.035 | 0.084±0.072 | 0.045±0.026 | 0.001±0.001 | 0.008 | 0.010 | 0.027 | 0.034 | 0.043 | 0.170 |
| angu | 0.101±0.056 | 0.097±0.058 | 0.088±0.043 | 0.089±0.042 | 0.086±0.075 | 0.034±0.024 | 0.011±0.006 | 0.000±0.000 | 0.005 | 0.025 | 0.028 | 0.044 | 0.173 |
| prod | 0.104±0.045 | 0.099±0.046 | 0.090±0.036 | 0.089±0.038 | 0.087±0.071 | 0.041±0.024 | 0.014±0.006 | 0.007±0.000 | 0.001±0.001 | 0.023 | 0.028 | 0.046 | 0.175 |
| aust | 0.094±0.044 | 0.089±0.046 | 0.084±0.035 | 0.081±0.033 | 0.079±0.057 | 0.038±0.022 | 0.036±0.018 | 0.033±0.019 | 0.032±0.016 | 0.002±0.001 | 0.021 | 0.044 | 0.165 |
| hubb | 0.089±0.039 | 0.086±0.040 | 0.086±0.032 | 0.077±0.025 | 0.081±0.037 | 0.045±0.022 | 0.049±0.022 | 0.042±0.021 | 0.042±0.019 | 0.034±0.015 | 0.011±0.003 | 0.043 | 0.183 |
| bili | 0.097±0.038 | 0.093±0.039 | 0.082±0.029 | 0.079±0.029 | 0.079±0.058 | 0.065±0.034 | 0.055±0.023 | 0.057±0.029 | 0.059±0.025 | 0.058±0.025 | 0.060±0.024 | 0.001±0.001 | 0.183 |
| Gmor | 0.184±0.159 | 0.188±0.018 | 0.178±0.124 | 0.169±0.091 | 0.181±0.000 | 0.188±0.176 | 0.181±0.167 | 0.183±0.171 | 0.185±0.165 | 0.178±0.165 | 0.196±0.098 | 0.192±0.152 | 0.000±0.000 |

**Supplementary Table S7.** Intraspecific nucleotide diversity of ITS1Nes sequences (*P_i_* ± S.D., on the diagonal), average number of nucleotide substitutions per site between species (*D*_xy_ ± S.D., below the diagonal) as generated with DnaSP v5.10.1 ^[78]^ and Net Value of Evolutionary Divergence between species (*d*, above the diagonal) as generated with MEGA v7.0.20 ^[77]^. Dotted lines separate pairwise comparisons within and between hake species (lowercase codes, Table 1) and morphotypes (uppercase codes, Table 2). The species *M. merluccius* is included as a relative measure of divergence.

|  | merl | prod | gayi | angu | aust | hubb | bili | albi | AUST | TASM | POLY | PATA | HUBB |
| --- | --- | --- | --- | --- | --- | --- | --- | --- | --- | --- | --- | --- | --- |
| merl | 0.000±0.000 | 0.128 | 0.128 | 0.134 | 0.131 | 0.182 | 0.135 | 0.144 | 0.151 | 0.116 | 0.126 | 0.165 | 0.136 |
| prod | 0.111±0.114 | 0.000±0.000 | 0.000 | 0.000 | 0.026 | 0.057 | 0.059 | 0.061 | 0.009 | 0.007 | 0.007 | 0.014 | 0.008 |
| gayi | 0.111±0.098 | 0.000±0.000 | 0.000±0.000 | 0.000 | 0.026 | 0.057 | 0.059 | 0.061 | 0.009 | 0.007 | 0.007 | 0.014 | 0.008 |
| angu | 0.115±0.102 | 0.000±0.000 | 0.000±0.000 | 0.000±0.000 | 0.027 | 0.059 | 0.044 | 0.060 | 0.009 | 0.008 | 0.008 | 0.015 | 0.009 |
| aust | 0.113±0.114 | 0.031±0.014 | 0.031±0.019 | 0.032±0.020 | 0.000±0.000 | 0.027 | 0.060 | 0.062 | 0.034 | 0.029 | 0.026 | 0.029 | 0.032 |
| hubb | 0.143±0.142 | 0.062±0.033 | 0.062±0.041 | 0.063±0.042 | 0.031±0.017 | 0.000±0.000 | 0.098 | 0.099 | 0.037 | 0.042 | 0.038 | 0.028 | 0.040 |
| bili | 0.117±0.116 | 0.066±0.033 | 0.066±0.043 | 0.050±0.032 | 0.067±0.035 | 0.100±0.059 | 0.000±0.000 | 0.000 | 0.070 | 0.040 | 0.041 | 0.076 | 0.053 |
| albi | 0.115±0.102 | 0.063±0.040 | 0.064±0.044 | 0.063±0.043 | 0.065±0.041 | 0.095±0.064 | 0.000±0.000 | 0.000±0.000 | 0.071 | 0.037 | 0.038 | 0.076 | 0.050 |
| AUST | 0.129±0.114 | 0.016±0.007 | 0.016±0.009 | 0.016±0.009 | 0.008±0.007 | 0.047±0.024 | 0.080±0.000 | 0.076±0.000 | 0.009±0.000 | 0.003 | 0.002 | 0.001 | 0.001 |
| TASM | 0.115±0.086 | 0.031±0.019 | 0.031±0.020 | 0.032±0.021 | 0.055±0.024 | 0.065±0.000 | 0.067±0.000 | 0.060±0.000 | 0.029±0.000 | 0.044±0.000 | 0.005 | 0.005 | 0.002 |
| POLY | 0.123±0.075 | 0.033±0.019 | 0.033±0.020 | 0.034±0.021 | 0.053±0.023 | 0.064±0.031 | 0.070±0.000 | 0.063±0.000 | 0.030±0.000 | 0.041±0.000 | 0.047±0.000 | 0.003 | 0.002 |
| PATA | 0.138±0.072 | 0.031±0.021 | 0.031±0.020 | 0.032±0.021 | 0.047±0.030 | 0.046±0.000 | 0.089±0.000 | 0.085±0.000 | 0.019±0.000 | 0.040±0.000 | 0.041±0.000 | 0.031±0.000 | 0.002 |
| HUBB | 0.124±0.097 | 0.028±0.012 | 0.028±0.013 | 0.028±0.014 | 0.054±0.018 | 0.061±0.000 | 0.075±0.000 | 0.068±0.000 | 0.022±0.000 | 0.037±0.000 | 0.039±0.000 | 0.033±0.000 | 0.034±0.000 |

**Supplementary Table S8. a)** Average number of nucleotide substitutions per site (*D*_xy_) and its standard deviation ($\bar{x}$ ± *S.D*.) between New World hakes (Pacific/Austral/Atlantic; lowercase codes, Table 1) and hake morphotypes (uppercase codes, Table 2), as generated with DnaSP v5.10.1 ^[78]^; **b)** Estimate of the Net Evolutionary Divergence (*d*) between New World hakes (Pacific/Austral/Atlantic) and hake morphotypes as generated with MEGA v7.0.20 ^[^^77]^. Both estimates were computed on ITS1Nes sequences. The European hake (merl) is included as a relative measure of divergence.

| **a** | prod | gayi | angu | $\bar{x}$±*S.D.*^1^ | CI |  | aust | hubb | $\bar{x}$±*S.D.* ^2^ | CI |  | bili | albi | $\bar{x}$±*S.D.* ^3^ | CI |
| --- | --- | --- | --- | --- | --- | --- | --- | --- | --- | --- | --- | --- | --- | --- | --- |
| AUST | 0.016 | 0.016 | 0.016 | 0.016±0.000 | [0.016, 0.016] |  | 0.008 | 0.047 | 0.019±0.028 | [0.008, 0.047] |  | 0.080 | 0.076 | 0.078±0.003 | [0.074, 0.082] |
| TASM | 0.031 | 0.031 | 0.032 | 0.031±0.001 | [0.031, 0.032] |  | 0.055 | 0.065 | 0.060±0.007 | [0.055, 0.065] |  | 0.067 | 0.060 | 0.064±0.005 | [0.057, 0.070] |
| POLY | 0.033 | 0.033 | 0.034 | 0.033±0.001 | [0.033, 0.034] |  | 0.053 | 0.064 | 0.058±0.007 | [0.053, 0.064] |  | 0.070 | 0.063 | 0.066±0.005 | [0.063, 0.070] |
| PATA | 0.031 | 0.031 | 0.032 | 0.031±0.001 | [0.031, 0.032] |  | 0.047 | 0.046 | 0.046±0.001 | [0.046, 0.047] |  | 0.089 | 0.085 | 0.087±0.003 | [0.083, 0.091] |
| HUBB | 0.028 | 0.028 | 0.028 | 0.028±0.000 | [0.028, 0.028] |  | 0.054 | 0.061 | 0.057±0.005 | [0.051, 0.064] |  | 0.075 | 0.068 | 0.071±0.005 | [0.065 0.078] |
| merl | 0.111 | 0.111 | 0.115 | 0.112±0.002 | [0.111, 0.115] |  | 0.113 | 0.143 | 0.127±0.021 | [0.098, 0.157] |  | 0.117 | 0.115 | 0.116±0.001 | [0.115, 0118] |
| **b** | prod | gayi | angu | $\bar{x}$±*S.D.*^1^ | CI |  | aust | hubb | $\bar{x}$±*S.D.*^2^ | CI |  | bili | albi | $\bar{x}$±*S.D.*^3^ | CI |
| AUST | 0.009 | 0.009 | 0.009 | 0.009±0.000 | [0.009, 0.009] |  | 0.034 | 0.037 | 0.035±0.002 | [0.032, 0.038] |  | 0.070 | 0.071 | 0.070±0.001 | [0.069, 0.071] |
| TASM | 0.007 | 0.007 | 0.008 | 0.007±0.001 | [0.007, 0.008] |  | 0.041 | 0.042 | 0.041±0.001 | [0.041, 0.042] |  | 0.040 | 0.037 | 0.038±0.002 | [0.036, 0.041] |
| POLY | 0.007 | 0.007 | 0.008 | 0.007±0.001 | [0.007, 0.008] |  | 0.026 | 0.038 | 0.031±0.008 | [0.020, 0.043] |  | 0.041 | 0.038 | 0.039±0.002 | [0.036, 0.042] |
| PATA | 0.014 | 0.014 | 0.015 | 0.014±0.001 | [0.014, 0.015] |  | 0.029 | 0.028 | 0.028±0.001 | [0.028, 0.029] |  | 0.076 | 0.076 | 0.076±0.000 | [0.076, 0.076] |
| HUBB | 0.008 | 0.008 | 0.009 | 0.008±0.001 | [0.008, 0.009] |  | 0.032 | 0.040 | 0.036±0.006 | [0.028, 0.043] |  | 0.053 | 0.050 | 0.051±0.002 | [0.048, 0.054] |
| merl | 0.128 | 0.128 | 0.134 | 0.130**±**0.003 | [0.128, 0.134] |  | 0.131 | 0.182 | 0.154**±**0.036 | [0.104, 0.204] |  | 0.135 | 0.144 | 0.139**±**0.006 | [0.131, 0.148] |

**^1^** *D*_xy_ average (**a**) or *d* (**b**) on pairwise comparisons between Pacific hakes (*M. productus*, *M. gayi* and *M. angustimanus*) and hake morphotypes.

**^2^** *D*_xy_ average (**a**) or *d* (**b**) on pairwise comparisons between Austral hakes (*M. australis* and *M. hubbsi*) and hake morphotypes.

**^3^** *D*_xy_ average (**a**) or *d* (**b**) on pairwise comparisons between Atlantic North hakes (*M. bilinearis* and *M. albidus*) and hake morphotypes.

**Supplementary Table S9.** Origin of nine hake morphotypes from directed sampling campaigns and museum holotypes and paratypes used in molecular phylodiagnosis. Code UAB and Subcode UAB are the internal entries of samples and tissues (liver, L; muscle, M) from those specimens from the Universidad Autónoma de Barcelona; No. ITS1Nes sequences is the No. of clones sequenced per tissue.

| **Morphotypes** | **Code** | **Reference** | **Location** | **Coordinates** | **No. specimens (Code UAB)** | **Subcode UAB** | **Tissue** | **No. ITS1Nes sequences** |
| --- | --- | --- | --- | --- | --- | --- | --- | --- |
| *Merluccius tasmanicus* | TASM | Holotype NMNZ P.5566 ^[24]^ | Tasman Bay, New Zealand | 40º52'S / 173º08'E | 1 (M3) | M32  M31 | L  M | 3  3 |
| *Merluccius tasmanicus* | TASM | Paratype NMNZ P.3963 ^[24]^ | Cook Strait, New Zealand | 41º30'S174º30'E | 1 (M4) | M42  M41 | L  M | 1  - |
| *Merluccius patagonicus* | PATA | Paratypes IIPB 501-  504/2001 ^[23]^ | Comodoro Ribadavia, Argentina | 45º30'S / 65º30'W | 1 (M10)  1 (M11)  1 (M12) | M101  M102  M111  M121  M122 | M  L  M  M  L | 1  -  1  1  - |
| *Merluccius polylepis* | POLY | Holotype ^[32]^ | Chiloé, Chile | 41º20'S / 74º35'W | 1 (M1) | M12  M11 | L  M | 3  3 |
| *Merluccius polylepis* | POLY | Paratype USNM 157765 ^[23]^ | Puerto Montt, Chile | 41º57'S / 72º87'W | 1 (M2) | M21  M22 | M  L | -  2 |
| *Merluccius hubbsi* | HUBB | Paratype IIPB 92/1987 ^[23]^ | Beagle Channel, Argentina | 54°48. 9'S / 68°14.8'W | 1 (M9) | M91  M92 | M  L | 3  3 |
| *Merluccius hubbsi* | HUBB | Uncatalogued juveniles ^[23]^ (*cf.* A. E. Ruiz and R. R. Fontdacaro) | Puerto Madryn, Argentina | 43°50'S / 65°02'W | 1 (M13)  1 (M14) | M131  M132  M141  M142 | M  L  M  L | 7  3  2  2 |
| *Merluccius australis* | AUST | Paratypes MOVI 27492-27493, formerly NMNZ P.13122  ^[24]^ | Chalkey Intel, Fiordland, New Zealand | 46º03'S/166º20'E | 1 (M5)  1 (M6) | M51  M61 | M  M | 2  4 |
| *Merluccius australis* | AUST | Uncatalogued juveniles ^[23]^ (*cf.* R. Bravo) | Aysén, Chile | 46º22’S/ 75º27’W | 1 (M7)  1 (M8) | M72  M71  M82  M81 | L  M  L  M | 1  -  -  1 |
